# Toll-Like Receptor Evolution: Does Temperature Matter?

**DOI:** 10.3389/fimmu.2022.812890

**Published:** 2022-02-14

**Authors:** Cármen Sousa, Stefan A. Fernandes, João C. R. Cardoso, Ying Wang, Wanying Zhai, Pedro M. Guerreiro, Liangbiao Chen, Adelino V. M. Canário, Deborah M. Power

**Affiliations:** ^1^ Centro de Ciências do Mar (CCMAR), Universidade do Algarve, Faro, Portugal; ^2^ International Research Center for Marine Biosciences, Ministry of Science and Technology, Shanghai Ocean University (SHOU), Shanghai, China

**Keywords:** TLR, Antarctic fish, innate immunity, immune challenge, cold temperature, evolution

## Abstract

Toll-like receptors (TLRs) recognize conserved pathogen-associated molecular patterns (PAMPs) and are an ancient and well-conserved group of pattern recognition receptors (PRRs). The isolation of the Antarctic continent and its unique teleost fish and microbiota prompted the present investigation into Tlr evolution. Gene homologues of *tlr* members in teleosts from temperate regions were present in the genome of Antarctic Nototheniidae and the non-Antarctic sister lineage Bovichtidae. Overall, in Nototheniidae apart from *D. mawsoni*, no major *tlr* gene family expansion or contraction occurred. Instead, lineage and species-specific changes in the ectodomain and LRR of Tlrs occurred, particularly in the Tlr11 superfamily that is well represented in fish. Positive selective pressure and associated sequence modifications in the TLR ectodomain and within the leucine-rich repeats (LRR), important for pathogen recognition, occurred in Tlr5, Tlr8, Tlr13, Tlr21, Tlr22, and Tlr23 presumably associated with the unique Antarctic microbiota. Exposure to lipopolysaccharide (*Escherichia coli* O111:B4) Gram negative bacteria did not modify *tlr* gene expression in *N. rossii* head–kidney or anterior intestine, although increased water temperature (+4°C) had a significant effect.

## 1 Introduction

The innate immune system is a defense mechanism present in all metazoans and provides a rapid and nonspecific cellular and humoral response against a wide range of viruses, bacteria, fungi, and parasites (1). The molecular elements of innate immunity include soluble mediators (such as cytokines, chemokines, antimicrobial peptides, lytic enzymes, and growth inhibitors) and innate immune cells such as granulocytes and phagocytes and innate immune host-cell receptors (2, 3). Tissues such as the thymus and head–kidney (primary immune organs) and the spleen, liver, and mucosa-associated lymphoid tissues, such as the skin and intestine (secondary immune organs), play a major role in innate immunity (4). The antigen-specific acquired immune response involving B and T lymphocytes is poorly developed in fish and so innate immunity is proposed as the prevalent defense mechanism (2, 3, 5).

Innate immune host-cell receptors detect beneficial and pathogenic microorganisms through the recognition of microbe-associated molecular patterns (MAMPs) and pathogen-associated molecular patterns [PAMPs (6)]. A broad repertoire of pattern recognition receptors (PRRs) is encoded in the germline of invertebrate and vertebrate host cells and includes Toll-like-receptors (TLRs), C-type lectin receptors, RIG-I-like receptors, and NOD-like receptors that survey the host microbiome (7, 8). The TLR superfamily of PRRs are transmembrane (TM) type-I glycoproteins best characterized for their interaction with PAMPs and modulation of host innate immunity through the NF-κB signaling pathway (9–11). A diverse range of pathogen-derived macromolecules (proteins, lipids, carbohydrates, and nucleic acids) are recognized by the extracellular N-terminal leucine-rich repeat (LRR) motif of TLRs, which is under high selective pressure for pathogen recognition (12, 13). In mammals, TLR activation can also be triggered by non-pathogenic host factors such as tri-acyl peptides, mRNA, and heat-shock proteins (14, 15), but whether this occurs in other vertebrates, including teleost fish, remains unknown (16–22). TLRs are also characterized by a highly conserved Toll/interleukin-1 receptor (TIR) intracellular domain that, after receptor interaction with PAMPs, triggers the intracellular signaling cascade that leads to an inflammatory response (23–28).

In humans, 10 well-characterized TLRs (TLR1–TLR10) exist, and in other vertebrates, the gene number is variable. In teleost fishes, the most successful and diverse group of vertebrates, up to 16 Tlrs, have been reported but gene number varies across species (29–31). Exceptions occur in the cod (*Gadus morhua*) and the zebrafish (*Danio rerio*), which underwent isoform-specific gene family expansions to generate 43 and 24 *tlr*, respectively (32). The acquisition of extra *tlr* isoforms in cod has been correlated with the highly variable pathogen loads and community composition of the paleoclimatic Arctic conditions (32). In fact, the large number and diversity of bacteria and viruses found in aquatic environments is proposed to have strongly influenced the evolution of innate immunity in teleosts (33). Tlr function in immunity is largely unexplored in teleost fish but protein homology indicates they probably recognize similar pathogens to TLR in mammals, although some can recognize both bacterial and viral pathogens (24, 31).

Here we test the hypothesis that Nototheniidae (Perciformes order, Notothenioidei sub-order), a stenothermal monophyletic teleost clade that evolved relatively recently in the cold-stable waters of Antarctica (34), and are exposed to a unique microbiota, possess a divergent *tlr* gene repertoire compared to other vertebrates. This species-rich fish lineage arose through adaptive radiation approximately 14–22 million years ago and compared to the non-Antarctic sister lineage (Bovichtidae family) acquired specific morphological and molecular modifications driven by the extreme cold and stable sea water environment (range ca. −2°C to +2°C) (35). The impact of the Nototheniidae species radiation and environmental adaptation on immune system evolution, which is hypothesized to drive teleost speciation and success, remains poorly described. As a first approach to evaluate the immune response of Antarctic fish, *Notothenia rossii* was challenged with a commonly used and commercially available bacterial endotoxin, LPS (*E. coli* O111:B4), and *tlr* gene expression and the activity of two plasma enzymatic markers of innate immunity were assessed in plasma of fish maintained at normal and increased seawater temperatures.

## 2 Materials and Methods

### 2.1 *In Silico* Database Searches

To characterize *tlr* gene members in Notothenioidei, available molecular data for six species of the Nototheniidae family *Notothenia coriiceps*, *Chionodracos hamatus*, *Trematomus bernacchii*, *Pseudochaenichthys georgianus*, *Gymnodraco acuticeps*, and *Dissostichus mawsoni*, and *Cottoperca gobio* of the sub-Antarctic Bovichtidae family were screened for homologues using the previously reported *N. coriiceps* sequences (23) as the query using BLASTp (36). To increase the number of Nototheniids in the analysis, an “in-house” *de novo* multi-tissue transcriptome assembly of *N. rossii* was also interrogated (**Supplementary Table 1**) and the retrieved sequences were translated into predicted proteins with the ExPASy translation tool (37) and their identity was confirmed by searching against the human sub dataset [human (taxid:9606)] of the non-redundant (nr) nucleotide database at the NCBI. The *tlr* gene complement in the genomes of seventeen other teleosts were also determined and included other Perciformes (same order as Nototheniidae), *Gasterosteus aculeatus* (Gasterosteidae family), and two other evolutionary proximate species within the Eupercaria clade, *Dicentrarchus labrax* (Moronidae family) and *Sparus aurata* (Sparidae family) (38). Representatives of other teleost taxonomic orders were also included in the analysis, Beloniformes (*Oryzias latipes*), Characiformes (*Astyanax mexicanus*), Cichliformes (*Oreochromis niloticus*), Cyprinodontiformes (*Poecillia formosa* and *Xiphophorus maculatus*), Cypriniformes (*D. rerio*), Gadiformes (*G. morhua*), Pleuronectiformes (*Paralychthys olivaceus*, *Cynoglossus semilaevis*, *Solea senegalensis*, and *Hippoglossus hippoglossus*), Salmoniformes (*Salmo salar*), and Tetraodontiformes (*Takifugu rubripes* and *Tetraodon nigroviridis*) (38). Putative *tlr* genes were retrieved based on their high sequence homologies (cutoff e value < e^-40^) with the query sequences and database orthologue/paralogue genome annotations (**Supplementary Table 1**).

To better understand *tlr* gene evolution in fish, the genomes of early diverging fish species such as the lobe-finned fish, *Latimeria chalumnae*, the ray-finned fish, *Lepisosteus oculatus*, two cartilaginous fish, *Callorhinchus milii* and *Rhincodon typus*, and the agnathan, *Petromyzon marinus*, were also interrogated for *tlr* genes. All searches were performed against the most recently annotated fish genome assemblies available from ENSEMBL or NCBI (**Supplementary Table 1**). The deduced Tlr amino acid sequences from *T. bernacchii* and *N. coriiceps* were used to retrieve homologue genes from the genomes of 5 tetrapods in ENSEMBL (*Homo sapiens*, *Mus musculus*, *Gallus gallus*, *Anolis carolinensis*, and *Xenopus tropicalis*), which were used for comparative analysis.

#### 2.1.1 Sequence Comparisons and Phylogenetic Analysis

The deduced Nototheniidae Tlr protein sequences were compared with the homologues from other species. Protein sequences were aligned with the MUSCLE algorithm available from the Aliview platform v1.22 (39) and the percentage of amino acid sequence identity/similarity was calculated using GeneDoc v2.7 software. The localization of protein domains characteristic of TLRs such as the LRR (typical LRR conserved motif, LxxLxLxxNxL, where x represents any aa), TM, and TIR motifs were predicted using ScanProsite (40), TMHMM Server v. 2.0 (http://www.cbs.dtu.dk/services/TMHMM/) and the Simple Modular Architecture Research Tool (SMART) (41) and also by the identification of highly homologous regions in the aligned sequences. The presence of a signal peptide was predicted using the SignalP 4.1 Server (42).

For the phylogenetic analysis, short incomplete sequences were removed from the multiple protein sequence alignment (**Supplementary Table 1**). Very similar sequences retrieved from the cod genome and that resulted from tandem gene duplications were also removed. The protein sequence alignment was manually edited to remove large gaps and misaligned sequences and the edited alignment containing the three main TLR protein domains (LRR, TM, and TIR) was used for the construction of phylogenetic trees using Bayesian inference (BI) and maximum likelihood (ML) methods. The dataset used to construct both trees was based on an alignment of 431 sequences. Phylogenetic trees were constructed using a VT model since it best fit the data given by model test-ng 0.1.5 in the CIPRES Science Gateway v3.3 (43). The BI tree was built using MrBayes (44) and 1,000,000 generation sampling and probability values to support tree branching. The ML tree was built using the RAxML v8.2.12 (45) method with 1,000 bootstrap replicates. Three cnidarian TLR-like sequences [obtained from (31)] were used to root the trees. The BI and ML trees were visualized in FigTree v1.4.3 (46) and edited using Inkscape v0.92.3.

#### 2.1.2 Short-Range Gene-Linkage Analysis

To better characterize the evolution of *tlr* genes in Notothenioidei and confirm if gene absence in Antarctic fish genomes was due to technical issues linked to genome assembly the result of gene loss, the localization of five to seven genes in the neighborhood of *tlr1*, *tlr2a* and *b*, *tlr5* and *tlr5S*, *tlr8*, and *tlr23* loci was characterized and compared across the four Nototheniidae representatives (*P. georgianus*, *N. coriiceps*, *T. bernacchii*, and *D. mawsoni*) and the sub-Antarctic *C. gobio* and *G. aculeatus.* Two Pleuronectiformes (*P. olivaceus* and *C. semilaevis*) were also included in the analysis as the genomes of this teleost order possess a reduced *tlr* gene number in relation to other teleosts. The flanking genome regions of *tlr1*, *tlr2*, *tlr5*, *tlr5S*, and *tlr8* characterized in *G. aculeatus* and in *T. bernacchii* were used as the reference to identify homologue genome regions in other species. For *tlr23*, the flanking genes of *P. georgianus* and *N. coriiceps* were used. The criteria for species selection were (a) the presence of the target genes and (b) the quality of the genome assemblies. Neighboring genes were identified using NCBI/ENSEMBL genome annotations or by sequence similarity searches against the genome assembly of each species.

#### 2.1.3 Selective Pressure Analysis

Antarctic fish Tlr amino acid sequences were aligned with the sub-Antarctic *C. gobio*, *G. aculeatus*, *S. aurata*, *D. labrax*, and the Pleuronectiformes (*P. olivaceus*, *C. semilaevis*, *S. senegalensis*, and *H. hippoglossus*) orthologues using the Aliview platform v1.22 (39) with the default settings. To obtain multiple codon alignments, PAL2NAL v14 (47) was used with the gap removal option and Neighbor-Joining trees for each gene family were built in MEGA X v10.1.8 (48) with 1,000 bootstrap replicates. Tlr sequences that were incomplete were removed from the analysis. To identify potential sequence changes that might be associated with the adaptation to the Antarctic environment, a branch-site analysis (BSM) was performed for each Tlr on the ancestral branch of Antarctic fish. For the specific case of *tlr21* the branch-site analysis was performed on each *G. acuticeps* duplicate. A sites analysis (SM) was performed with the sequences of the Antarctic species if positive selection was identified on their ancestral branch.

The branch-site and sites analysis used to test for evolutionary pressure were based on the ML method of the Codeml [PAML v4.9 package (49)] program in EasyCodeml (50). Codon substitution models were compared with likelihood ratio tests (LRT) calculated from the difference between their log likelihood (lnL) values.

### 2.2 Experimental Immune Challenge

Animal collection and experimentation were approved by the Portuguese Environment Agency, under the regulations set by the Treaty of Madrid for scientific investigation in Antarctica. The experiments performed complied with EU and Portuguese regulations for animal experimentation.

Adult *N. rossii* (30 ± 4.2 cm length and 312 ± 124.7 g weight) were captured (depths 10–30 m) using a hook-and-line in the Antarctic Peninsula, near the Great Wall Station (GPS coordinates: 62°13′″S, 58°58′W) located in King George Island. Captured fish were maintained in a flow-through seawater circuit in 200-L plastic tanks for up to 5 days before the immune challenge. Fish were fed twice a day (morning and evening) with a mixture of limpets, salps, amphipods, and small fish. No mortality was observed during acclimation or during the experiments.

The experimental immune challenge was performed between January and February of 2019, during the Antarctic summer when the seawater temperature is ±2°C. For the immune challenge, fish were lightly anesthetized in 2-phenoxyethanol (0.1 ml/L, Sigma-Aldrich) and the control fish (*n* = 6) were injected intraperitoneally (IP) with saline buffer [0.2% (v/w) of 1.1% NaCl]; the immune challenged group (*n* = 6) was injected with 1.5 mg/ml LPS (extracted from *Escherichia coli* O111:B4, L2630, Sigma-Aldrich, Portugal) dissolved in saline buffer (**Supplementary Figure 1**). LPS was chosen as a proxy for a bacteria-like immune challenge because it is reported to play a key role in host pathogen interactions and has previously been shown to modify *tlr* gene expression in teleost fish (51–53). The dose of LPS and route of administration were selected based on previous studies in teleost fish (54–59). Water temperature (2.1 ± 0.5°C), salinity (29 ± 0.5 ppt), and oxygen levels (10.7 ± 0.4 ppm) were monitored three times a day (7 a.m., 2 p.m., and 9 p.m.). To assess the impact of water temperature on the immune response of *N. rossii*, a similar immune challenge experiment was run in parallel at 6.0 ± 0.8°C; all other parameters were the same except the oxygen levels that were slightly lower (9.3 ± 0.9 ppm) (**Supplementary Figure 1**). Fish were acclimatized by increasing the seawater temperature in the experimental tanks daily by 1.5°C from 2 to 6°C.

Fish were euthanized 8 h and 24 h post-IP injection with an overdose of 2-phenoxyethanol (1 ml/L, Sigma-Aldrich), weighed, and kept on ice under atmospheric ambient temperature (2°C) for blood and tissue sampling. The blood was collected from the caudal vasculature using a heparinized 1-ml syringe fitted with a 21-gauge needle. Blood was centrifuged at 10,000 *g*, and 4°C for 4 min and the plasma was collected and stored at -80°C until analysis. For tissue sample collection the fish were decapitated and then the head–kidney (principal hematopoietic organ in teleosts) and anterior intestine (duodenum region) were dissected out and placed in RNAlater (Sigma-Aldrich) for 24 h at 4ºC before storage at -20°C.

### 2.3 RNA Extraction, cDNA Synthesis, and Quantitative PCR Analysis

Total RNA was extracted from *N. rossii* tissues (~25 mg) using an E.Z.N.A. Total RNA Kit I (Omega Bio-Tek, USA) and following the manufacturer’s instructions. The DNase I digestion protocol was performed directly on the columns and contaminating genomic DNA eliminated with RNase-free DNase I (Omega Bio-Tek). The integrity of the extracted RNA was evaluated by 1% agarose gel electrophoresis and the quantity and quality of the RNA was assessed by absorbance using a NanoDrop One (Thermofisher, Spain). Approximately 500 ng of *N. rossii* DNase-treated total RNA was used to synthesize cDNA in a final reaction volume of 20 µl with 200 ng of random hexamers (Jena Biosciences, Germany), 10 mM dNTPs (Promega, USA), 100 U RevertAid reverse transcriptase (RT, Promega), and 8 U of Ribolock RNase Inhibitor (ThermoFisher) for 10 min at 20°C, 50 min at 42°C, and 5 min at 72°C.

The expression of *tlr5*, *tlr21*, *tlr22*, and *tlr25* in *N. rossii* was determined by real-time quantitative PCR (RT-qPCR) using gene-specific primers. The candidate *tlr* gene transcripts analyzed were selected because they were the most abundant in “in-house” immune-related multi-tissue (head–kidney, skin, and intestine) transcriptomes of *N. coriiceps*. The reference genes selected for normalization, beta-actin (*β-actin*, TR9194|c3_g4_i8) and *18s* rRNA did not vary in expression between any of the *N. rossii* experimental groups. The cDNAs used for the RT-qPCR were diluted to a final concentration of 25 ng/µl for the candidate genes and to 10 ng/µl for *β-actin* and 0.01 ng/µl for *18s.* The qPCR reactions were performed in 96-well plates (Axygen, Germany) using a CFX96 Touch Real-Time PCR Detection System (Bio-Rad, USA). The final RT-qPCR reaction volume was 10 µl and contained 2 µl of cDNA, 5 µl of SsoFast Evagreen Supermix (Bio-Rad), and 0.3 mM of the forward and reverse specific primers (**Supplementary Table 2**). The thermocycle used was 95°C for 30 s, followed by 40 cycles of 95°C for 5 s and 10 s at the annealing temperature. Melting curves 60°C to 95°C with an increment of 0.5°C for each 10 s were performed to detect reaction specificity. All RT-qPCR analysis included a no template control and a -RT control. PCR efficiencies and the coefficient of determination (*r* ^2^) were calculated and were >90% for each target gene transcript analyzed. Gene expression levels were normalized using the geometric mean of the two reference genes (*18s* and *β-*actin) and the SQ mean of the target genes based on the standard curve method (60).

### 2.4 Blood Plasma Total Protein and Enzyme Activity

Total plasma protein was determined using a Quick Start ™ Bradford Protein Assay kit (Bio-Rad, Portugal) (61) adapted for a 96-well plate and reactions were analyzed at 590 nm at 25°C using a spectrophotometer (Agilent Technologies, USA).

The activity of lysozyme was measured using a turbidimetric assay (62). Briefly, 130 µl of lyophilized *Micrococcus luteus* cells (0.6 mg/ml, Sigma-Aldrich) in 0.05 M sodium phosphate buffer, pH 6.2, was mixed with 20 µl of blood plasma in a flat-bottomed 96 well-plate. For the standard curve, a concentration range from 50 U/ml to 500 U/ml of hen egg white lysozyme (Sigma-Aldrich) was used. All reactions were performed in duplicate and incubated at 25°C for 10 min and then measured in a multiplate reader (Agilent Technologies) at 450 nm.

Antitrypsin activity was used to assess total antiprotease activity (63). Trypsin from porcine pancreas (20 µl from a 5 mg/ml solution, Sigma-Aldrich) was mixed with *N. rossii* blood plasma (10 µl) for 10 min and then 200 µl of 0.1 M phosphate buffer pH 7.0 and 250 µl of 2% Azocasein (Sigma-Aldrich, Germany) was added. Reactions were performed in duplicate and were incubated for 1 h at 37°C. Subsequently, 500 µl of 10% trichloroacetic acid (Sigma-Aldrich) was added to the reaction and it was incubated for a further 30 min at room temperature (21°C) and then the samples were centrifuged at 10,000 rpm for 10 min. One hundred microliters of the supernatant from each of the reactions was transferred to a 96-well plate and 100 µl of sodium hydroxide (1N, NaOH, VWR, Spain) was added. Assays were measured in a multiplate reader (Agilent Technologies) at 450 nm.

### 2.5 Statistical Analysis

Differences in gene expression and enzyme activity between the control and LPS-treated groups were assessed at 8 h and 24 h after exposure and at different experimental seawater temperatures (+2°C and +6°C) using a three-way analysis of variance and post-hoc Tukey´s test (Shapiro–Wilk normality test). Graphs for the results of RT-qPCR were generated using SigmaPlot v12.5, and for enzymatic activity, GraphPrism v6.01 was used. Statistical significance was considered at *p* < 0.05.

## 3 Results

### 3.1 Bioinformatic Search for *tlrs* in Antarctic Notothenioids and Other Fish

**Figure 1:**
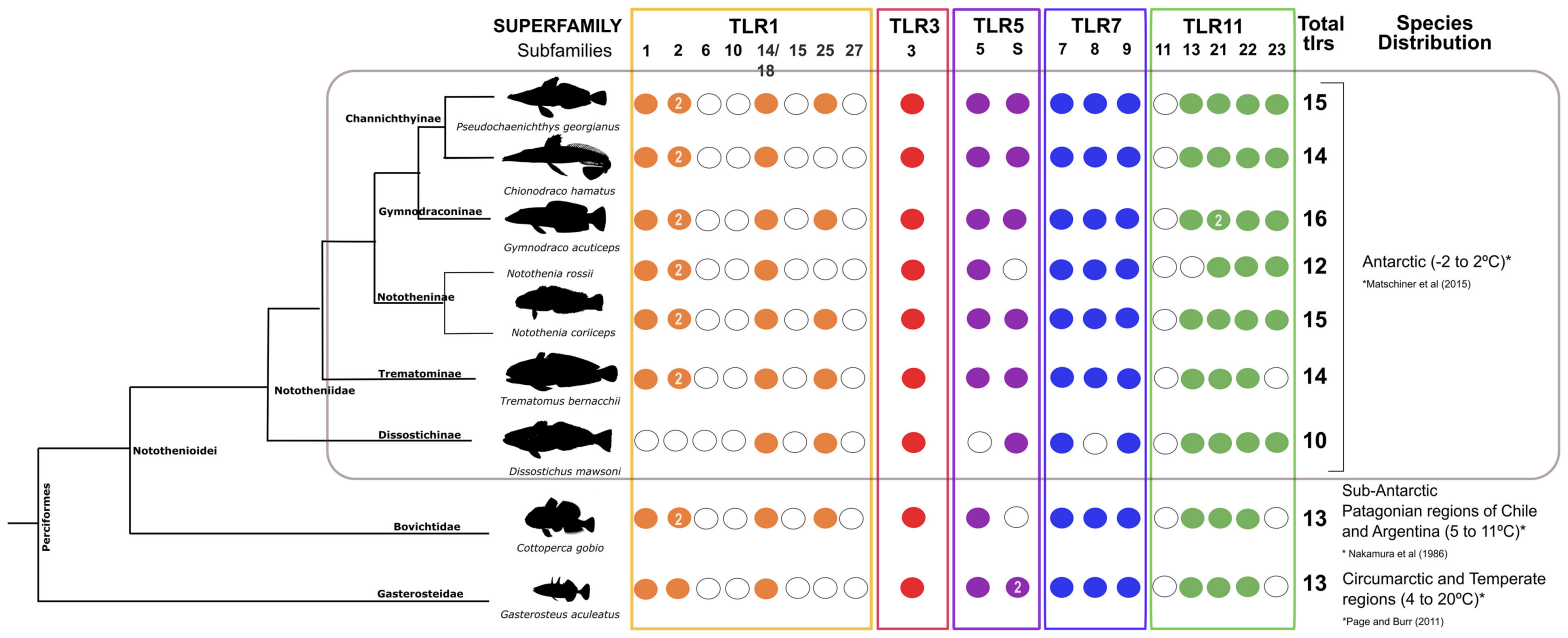
Detailed dendrogram of the *tlr* genes/transcripts in Nototheniidae. Genes/transcripts identified in Nototheniidae were distributed within five vertebrate TLR superfamilies and are indicated by the colored circles. When multiple genes were identified for a given *tlr* isoform, gene number is indicated inside the colored circles. Genes that were not identified are indicated with a white circle. *C. gobio* was used as a representative of the Nototheniidae sister lineage and *G. aculeatus* was used as a representative of the other Perciform species outside the Notothenioidei sub-order. *Tlr* gene family members from all species analyzed were obtained by searching their genome assemblies. The exception was *N. rossii* where an “in house” *de novo* multi-tissue transcriptome assembly was used. The figure was drawn considering the relative evolutionary relationship between the six Antarctic fish species and the sub-Antarctic fish (35, 64). Gene/transcript accession numbers are available in **Supplementary Table 1**.

Ten *TLR* (*TLR1* to *TLR10*) genes exist in humans (*H. sapiens*), and sequence similarity of the deduced proteins clusters them in six superfamilies containing various members: TLR1 (TLR1, TLR2, TLR6, and TLR10), TLR3 (TLR3), TLR4 (TLR4), TLR5 (TLR5 and TLR5S), and TLR7 (TLR7, TLR8, and TLR9). In fish, homologues of the human TLRs and of the non-mammalian TLR11 (TLR11, TLR13, TLR21, TLR22, and TLR23) superfamily were found (**Supplementary Figure 2**). Genes encoding homologues of human TLR1 to TLR3, TLR5, and TLR7 to TLR9 proteins were found in most fish genomes analyzed and in the teleosts gene number varied from 11 to 16 except in *D. rerio* (Cypriniformes) and *G. morhua* (Gadiformes) where 24 and 42 genes, respectively, were retrieved (**Supplementary Figure 2**). The Pleuronectiformes had less *tlr* genes than other teleosts and *S. senegalensis* and *C. semilaevis* had 6 and 7 genes, respectively (**Supplementary** **Figure 3**). Sequence searches in six Nototheniidae revealed that they have a similar gene number to other teleosts (**Supplementary Figure 2** and **Figure 1**). *D. mawsoni* had the lowest number of *tlr* genes (10) and *G. acuticeps* had the highest number of *tlr* genes (16). Although a genome is not available, analysis of a mixed tissue transcriptome of *N. rossii* revealed 12 *tlr* gene transcripts while in the genome of the evolutionary proximate species, *N. coriiceps*, 15 *tlr* genes were retrieved (**Figure 1**). In the *C. gobio* genome, a sub-Antarctic species, 13 *tlrs* were identified and *tlr5S* and *tlr23* genes were absent. In all Notothenioidei (except *D. mawsoni*) in common with other teleosts, two *tlr2* genes exist (**Figure 1**). In summary, there was no evidence of major gene family expansion or loss of TLR superfamilies in the Antarctic Nototheniidae. Retention of subfamily members was very similar in Nototheniidae except for *D. mawsoni* that specifically lost TLR1 and TLR7 superfamily members.

#### 3.1.1 Nototheniidae Contain a Similar *tlr* Gene Repertoire to Other Teleosts

**Figure 2:**
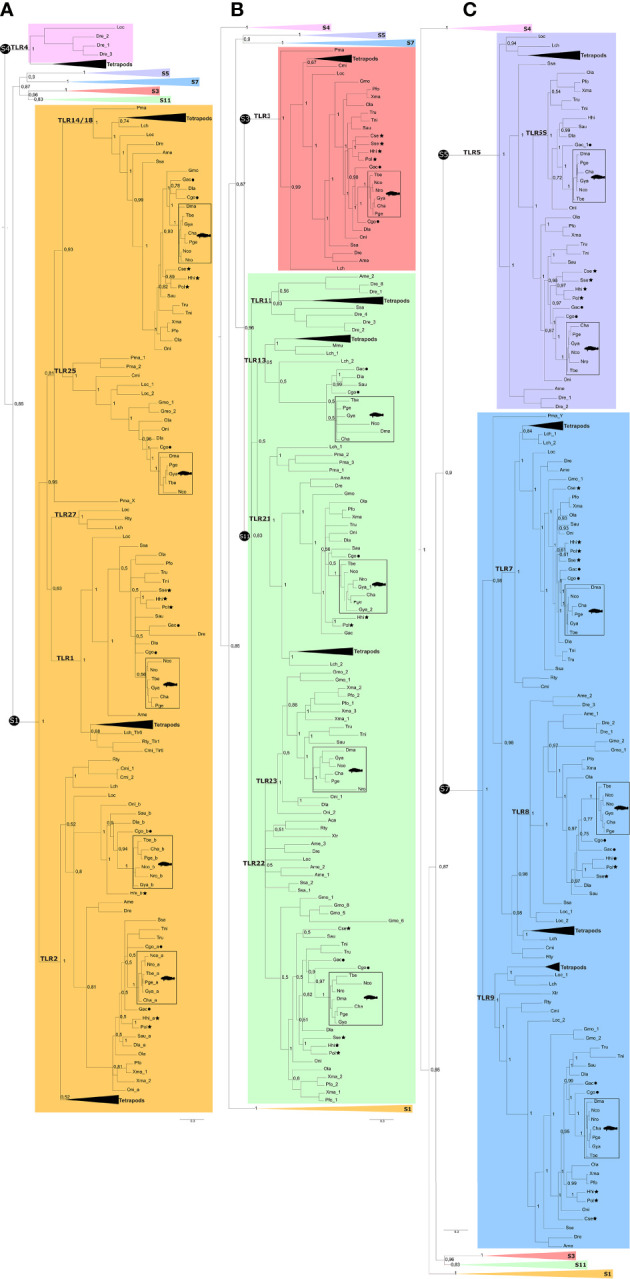
Simplified phylogenetic tree of the Tlrs from Nototheniidae and other vertebrates. The phylogenetic tree was constructed using the BI method and the full tree is available as **Supplementary Figure 3**. Branches corresponding to the six vertebrate TLR superfamilies are identified: S1 indicates the TLR1 superfamily (orange); S3 identifies the TLR3 superfamily (red); S4 identifies the TLR4 superfamily (pink); S5 identifies the TLR5 superfamily; S7 identifies the TLR7 superfamily (blue); and S11 identifies the TLR11 superfamily (green). Some branches of the phylogenetic tree are collapsed to facilitate visualization and three subsections of the same phylogenetic tree are shown: **(A)** the TLR1 superfamily, **(B)** the TLR11 and TLR3 superfamilies, and **(C)** the TLR5, TLR7, and TLR4 superfamilies. The tetrapod branches were also collapsed. The accession numbers of the sequences used to construct the phylogenetic tree are available in **Supplementary Table 1**. The teleost *tlr2* duplicates were named, *tlr2a* and *tlr2b*, and TLR15 was not included in the phylogenetic tree since it was only found in chicken and lizard. The phylogenetic tree was rooted with the Cnidarian Tlr clade (31). The sea lamprey *tlrs* within the vertebrate TLR1 and TLR7 superfamilies are named *Pma_X* and *Pma_Y*, respectively, since their assignment to the Tlr subfamilies was ambiguous. Only the posterior probability values for the main branches are indicated. A phylogenetic tree generated by the ML method is available as **Supplementary Figure 2**. Members from Antarctic species are indicated with squares and with a *Notothenia* cartoon to facilitate identification. The other member of the Perciform order, *G. aculeatus*, is indicated by a black dot and the Pleuronectiformes is indicated by a black star.

Phylogenetic analysis of the deduced Tlr proteins of Nototheniidae with other vertebrates confirmed the existence of receptors that belong to five TLR superfamilies (TLR1, TLR3, TLR5, TLR7, and TLR11) (**Figure 2** and **Supplementary Table 3**, **Supplementary Figures 4, 5**). The BI and ML trees share similar topologies and the Nototheniidae Tlrs cluster together and the Nototheniidae sequence branches were in most cases rooted with the representative of the sister-lineage, *C. gobio* (**Supplementary Figures 4, 5**).

##### 3.1.1.1 TLR1 and TLR4 Superfamilies

Members of the TLR1 superfamily were the most diverse in Nototheniidae as well as in other vertebrates. In contrast, TLR4 members were only present in tetrapods and in representatives of a few fish orders (Lepisosteiformes and the Cypriniformes) (**Figure 2A**). The TLR1 superfamily in Nototheniidae and other fish were grouped into two main clusters: one contained TLR1, TLR2, TLR6, TLR10, and TLR27 and the other contained TLR14/18 and TLR25 (**Figure 2A**). Clustering of the two *tlr2* genes found in Nototheniidae and in other teleost fish suggested that they were duplicates and arose during the teleost-specific genome duplication and the paralogues were named *tlr2*a and *tlr2b*. Members of the Tlr14/18 subfamily were confirmed in most fish orders but the *tlr25* gene was found in relatively few species (**Figure 2A**).

##### 3.1.1.2 TLR3 and TLR11 Superfamilies

A single *tlr3* gene (TLR3 superfamily) was present in Nototheniidae and in the other vertebrates. Members of the TLR11 superfamily were found in most fish including Nototheniidae (**Figure 2B**). The tree topology revealed that TLR11 superfamily members were grouped into two clusters: TLR11/TLR13/TLR21 and TLR22/TLR23. The BI and ML trees confirmed the absence of a *tlr11* homologue in Nototheniidae and most other teleosts (**Figure 2B**). Members of the TLR13 subfamily were only found in Nototheniidae, *G. aculeatus*, *S. aurata*, and *D. labrax* (all representatives of the Eupercaria clade).

*Tlr21* and *tlr22* genes were identified in fish and tetrapods but *tlr23* was exclusive to the fish. In Nototheniidae, all species possessed a single *tlr21*, *tlr22*, and *tlr23* gene except the Bathydraconidae, *G. acuticeps*, which had duplicate gene copies of *tlr21* (*tlr21_1* and *tlr21_2*). The *tlr23* gene was lost in *T. bernacchii.* The sub-Antarctic *C. gobio* also lacked the *tlr23* gene (**Figure 2B**). The phylogenetic trees confirmed the absence of *tlr21* not only from the Pleuronectiform, *C. semilaevis*, and the Salmoniform *S. salar* but also in the two cartilaginous fish analyzed (**Figure 2B**).

##### 3.1.1.3 TLR5 and TLR7 Superfamilies

The *tlr5* ancestral gene duplicated during the teleost radiation and originated TLR5 and TLR5S subfamilies (**Figure 2C**). Most of the Nototheniidae and other teleosts retained the two *tlr5* genes. In *D. mawsoni* (Dissostichus), only *tlr5S* was retained, and in the *C. gobio* (Bovichtidae), only *tlr5* persisted (**Figure 2C**). The Pleuronectiformes and the Gadiform *G. morhua* were an exception and generally lost *tlr5* and *tlr7*. The TLR7 superfamily gene precursor duplicated to originate the TLR7, TLR8, and TLR9 subfamilies (**Figure 2C**) of fish and tetrapods. Within the TLR7 superfamily, a single copy of *tlr7*, *tlr8*, and *tlr9* genes was found in most fish with few exceptions (**Figure 2C** and **Supplementary Table 1**). Clustering of the teleost sequences confirmed the absence of *tlr8* genes in the Antarctic *D. mawsoni* and in *T. rubripes* (Tetraodontiformes) and of *tlr9* from the Pleuronectiform *S. senegalensis* (**Figure 2C**).

#### 3.1.2 Species-Specific Gene Losses Occurred in the Nototheniidae, *D. mawsoni*

**Figure 3:**
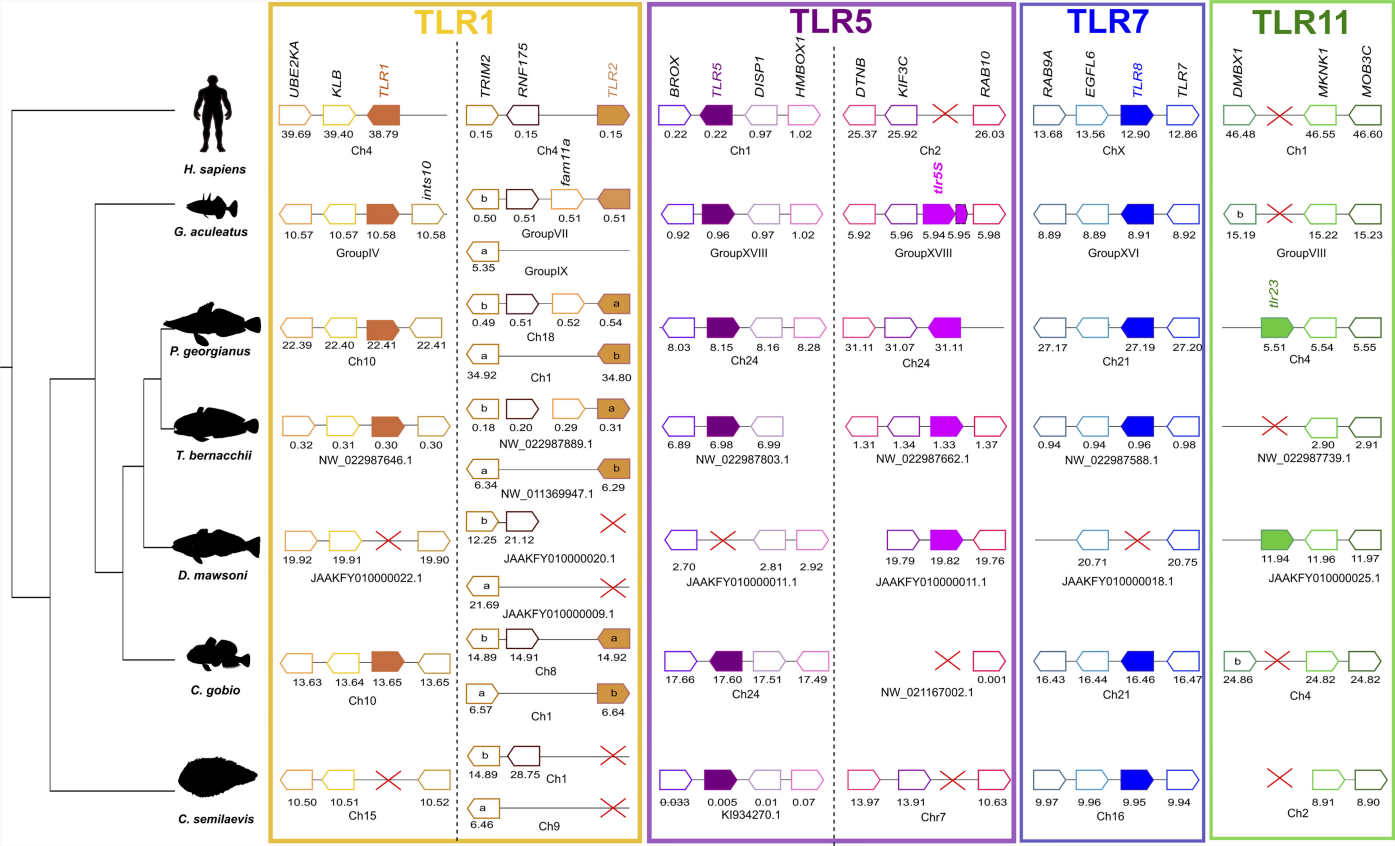
Gene synteny analysis of *tlr1*, *tlr2*, *tlr5*, *tlr5S, tlr8*, and *tlr23* in Antarctic Nototheniidae and other vertebrates. Presented are the Antarctic Nototheniidae *P. georgianus*, *T. bernacchii*, and *D. mawsoni*. Other vertebrates include *C. gobio* as the representative of the sister lineage, *G. aculeatus* as a non-Antarctic representative of the Perciform order, one representative of the Pleuronectiformes order, *C. semilaevis*, and *H. sapiens* as the tetrapod. Genome regions analyzed are indicated by a line and predicted genes are represented by arrows and the arrowhead indicates gene orientation and the gene symbol is given. The gene positions in the genome assemblies analyzed (Mega base pairs, Mbp) are indicated below each synteny map. *Tlr* genes are represented by full colored arrows. Extended analysis including more species are available as **Supplementary Figures 6–8**. The red cross indicates gene absence.

To understand *tlr* gene loss in the Nototheniidae, *D. mawsoni*, the neighboring gene environment was characterized. Overall, the results of the analysis indicated that gene loss in Nototheniidae and other teleosts was mainly due to species-specific gene deletion events (**Figure 3** and **Supplementary Figures 6–8**).

##### 3.1.2.1 Tlr1 Gene Linkage

In the *G. aculeatus* genome, *tlr1* mapped in group IV and five neighboring genes were identified (**Figure 3** and **Supplementary Figure 6**). In the *D. mawsoni* genome that lacks *tlr1*, the neighboring genes were identified on chromosome fragment JAAKFY010000022.1. This suggests that this genome region was highly conserved during evolution and that gene loss in *D. mawsoni* was a consequence of a species-specific gene deletion event (**Figure 3** and **Supplementary Figure 6**). A similar species-specific gene deletion occurred in the Pleuronectiform *C. semilaevis*.

##### 3.1.2.2 Tlr2 Gene Linkage

In teleosts including the Nototheniidae *tlr1* and *tlr2* genes mapped to different chromosomes or scaffolds (**Figure 3** and **Supplementary Figure 6**). In the *T. bernacchii* genome, the *tlr2a* gene mapped to NW_022987889.1 and *tlr2b* to NW_011369947.1. The flanking gene *trim2* was closely linked to *tlr2*, and *trim2b* shared the same genome region as *tlr2a* and *trim2a* was linked to *tlr2b* (**Figure 3** and **Supplementary Figure 6**) and most likely arose during the teleost specific genome duplication event.

In the *G. aculeatus* genome, a single *tlr2* gene mapped to group VII and the flanking genes shared synteny with *tlr2a* and a homologue genome region to *tlr2b* was identified in group IX, but *tlr2b* was absent, indicating that this gene was eliminated from the genome. In Pleuronectiformes, *tlr2a* mapped to *P. olivaceus* NW_017863846.1 but this scaffold was very short and only contained this gene. In *C. semilaevis* the *tlr2* gene duplicates were lost from chromosome 1 and chromosome 9 (**Figure 3** and **Supplementary Figure 6**).

##### 3.1.2.3 Tlr5 Gene Linkage

In *G. aculeatus* (group XVIII) and in the Nototheniidae, *P. georgianus* (chromosome 24) *tlr5* gene duplicates mapped to the same genome fragment (**Figure 3** and **Supplementary Figure 7**). In *G. aculeatus*, one of the *tlr5* genes was incomplete and may be a pseudogene. In the genome of the Nototheniidae, *T. bernacchii*, conserved homologue genome regions were found for both *tlr5* and *tlr5s* but they mapped to different genome fragments. In *D. mawsoni*, only *tlr5S* was identified and the flanking genes were conserved and mapped to JAAKFY010000011.1 along with the homologue genes flanking *tlr5* in other species (**Figure 3** and **Supplementary Figure 7**). In *C. gobio*, the *tlr5* gene and its conserved gene environment mapped to chromosome 24 but *tlr5S* was absent although the flanking genes were conserved (scaffold NW_021167002.1). In the two Pleuronectiformes, *P. olivaceus* and *C. semilaevis tlr5* were mapped to conserved genome regions in scaffolds NW_017859659.1 and KI934270.1, respectively. *Tlr5S* was lost but the genome region was conserved (**Figure 3** and **Supplementary Figure 7**).

##### 3.1.2.4 Tlr8 Gene Linkage

*Tlr8* mapped next to *tlr7* in group XVI of the *G. aculeatus* genome (**Figure 3** and **Supplementary Figure 8**) and gene synteny was conserved in all representatives of the Nototheniidae and in the sister lineage, the Bovichtidae (*C. gobio*). The exception was *D. mawsoni* in which a genome region containing *tlr7* was identified (JAAYFY010000018.1) but *tlr8* was absent (**Figure 3** and **Supplementary Figure 8**). In *H. sapiens*, TLR7 and TLR8 also map in proximity on chromosome X, suggesting that they probably arose through a tandem gene duplication prior to the teleost and tetrapod divergence.

##### 3.1.2.5 Tlr23 Gene Linkage

The *tlr23* gene mapped to chromosome 4 in *P. georgianus*, *D. mawsoni*, JAAKFY010000025.1, and NW_011369947.1 in the *N. coriiceps* genome (**Figure 3** and **Supplementary Figure 8**). In species where this gene was absent, a conserved gene environment was identified (*T. bernacchii*, NW_022987739.1; *C. gobio*, chromosome 4; *G. aculeatus*, group VIII; *P. olivaceus*, NW_017859646.1; and *C. semilaevis*, chromosome 2). This suggested that retention of the *tlr23* gene was species-specific in teleosts (**Figure 3** and **Supplementary Figure 8**). In the *H. sapiens* genome, no *tlr23* homologue was found although the genome region was conserved on chromosome 1.

#### 3.1.3 Nototheniidae Tlr Proteins Share Conserved Structure With Other Teleosts

Sequence comparisons of the deduced proteins revealed that they were relatively well conserved and shared between 70% and 98% amino acid (aa) similarity and the Nototheniidae Tlr3, Tlr5, and Tlr23 were the most conserved (91%–99% aa similarity). *C. gobio* Tlrs shared 64%–86% similarity with the Nototheniidae, and with other Perciformes, they were 48%–86% similar. All deduced Nototheniidae and *C. gobio tlr* sequences shared a conserved protein structure with the other teleosts and possessed several LRR motifs involved in pathogen recognition, and single TM and TIR domains. The exception was Tlr5S, which lacked the TM and the TIR domain in all teleost fishes and may be a soluble receptor isoform. The main difference between Tlr isoforms was the number of LRRs within the ectodomain, which was variable across the different subfamilies, and Antarctic fish possessed a similar number of LRRs to other Perciformes. In addition, the presence or absence of a predicted signal peptide in the gene members of the same subfamily suggested that functional divergence may exist between the homologue genes as they may have different cellular localizations (**Supplementary Figures 9–14**).

Members of Tlr8, Tlr9, and Tlr21 possessed the largest number of predicted LRR repeats at the ectodomain. They varied from 18 in most fishes to 19 in *C. gobio* and *T. bernacchii* Tlr8. For Tlr9, they varied from 16 in *G. aculeatus* to 20 in *C. hamatus* and *P. georgianus* and for Tlr21, 15 in *G. aculeat*us to 20 in *T. bernacchii*. The duplicate *G. acuticeps* Tlr21 genes shared a similar protein structure with 18 LRRs. The Tlr14/18 (LRRs 8–10) and Tlr25 (LRRs 7–8) possessed the least number of LRR repeats. The Tlr subfamilies with conserved LRR repeat number were Tlr1 (all species possessed 9 LRRs), Tlr8, and Tlr25. The Tlr subfamilies with the biggest LRR repeat variability were Tlr2a (8–12) and Tlr13 (10 to 15). Within the other superfamilies, the number of LRRs varied only by 2 LRR units (e.g., 13–15 for Tlr23 and 15–17 for Tlr3).

#### 3.1.4 Codon Usage Bias Potentially Modified Pathogen Recognition in Nototheniidae Tlr

**Figure 4:**
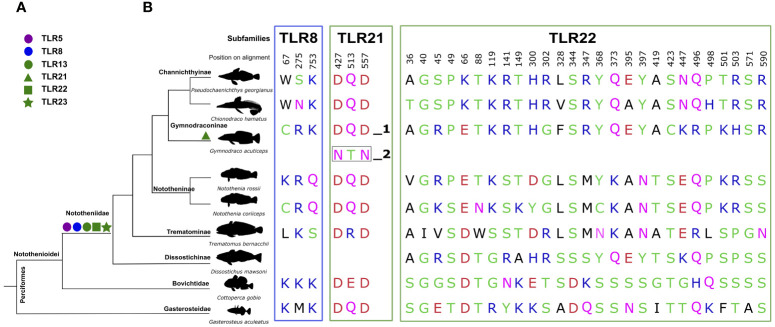
Evidence of positive selection in the LRR ectodomains of Nototheniidae Tlrs. Receptors represented are Tlr8 and Tlr21 and Tlr22. **(A)** Positive selection identified with the branch-site model is represented by colored shapes on the ancestral Nototheniidae branch and on the Tlr21 duplicate in *G. acuticeps* (Tlr21_2) and PSS were only found for Tlr21_2 and are boxed. **(B)** PSS identified with the branch-site and site models for Nototheniidae sequences. PSS were colored according to their physicochemical properties: acidic in red, basic in blue, neutral in purple, polar in green, and hydrophobic in black using Weblogo vs3 annotation. The amino acid positions given refer to the edited multiple sequence alignment used for the selective pressure analysis. The PSS represented were mapped in the sequences and are marked in red (**Supplementary Figures 4–8**).

Branch-site analysis for the ancestral Nototheniidae revealed that Tlr5 (BSM, *p* = 0.0011), Tlr8 (BSM, *p* < 0.001), Tlr13 (BSM, *p* = 0.0018), Tlr22 (BSM, *p* < 0.001), and Tlr23 (BSM, *p* = 0.001) were under positive selection (**Table 1**). Branch-site analysis was also performed for the duplicate *G. acuticeps* Tlr21_2 and revealed positive selection (BSM, *p* < 0.001) (**Table 1**). Site analysis was further performed for these receptors and identified several positive selection sites (PSS) within the ectodomain and inside the LRR motifs of Nototheniidae Tlr8, Tlr13, Tlr21, Tlr22, and Tlr23 (**Figure 4**) and resulted in species-specific modifications.

**Table 1:** Selective pressure analysis for the branch-site models.

Receptor	Model	np	Ln L	Estimates of parameters					Model compared	LRT P-value	Positive sites^a^
**TLR5**	Model A	28	-10165.09641	Site class	0	1	2a	2b	Model A vs.Model A null	0.001143784	530 N 0.978*
				f	0.60473	0.33234	0.04061	0.02232			
				ω0	0.10087	1	0.10087	1			
				ω1	0.10087	1	9.07143	9.07143			
	Model A null	27	-10170.38592	1							Not Allowed
**TLR8**	Model A	28	-16158.517734	Site class	0	1	2a	2b	Model A vs.Model A null	0.000231386	823 A 0.965*
				f	0.57462	0.40766	0.01037	0.00735			
				ω0	0.07029	1.00000	0.07029	1.00000			
				ω1	0.07029	1.00000	998.99874	998.99874			
	Model A null	27	-16165.296401	1							Not Allowed
**TLR13**	Model A	20	-7100.573648	Site class	0	1	2a	2b	Model A vs.Model A null	0.001826836	
				f	0.64259	0.32938	0.01853	0.00950			
				ω0	0.06914	1.00000	0.06914	1.00000			
				ω1	0.06914	1.00000	14.37178	14.37178			
	Model A null	19	-7105.431583	1							Not Allowed
**TLR22**	Model A	32	-17590.839397	Site class	0	1	2a	2b	Model A vs.Model A null	0.000001954	344 S 0.962*,627 M 0.960*
				f	0.49499	0.44812	0.02986	0.02703			
				ω0	0.13232	1.00000	0.13232	1.00000			
				ω1	0.13232	1.00000	12.14526	12.14526			
	Model A null	31	-17602.159433	1							Not Allowed
**TLR23**	Model A	16	-9143.957160	Site class	0	1	2a	2b	Model A vs.Model A null	0.009946886	Not significant sites
				f	0.56141	0.41365	0.01436	0.01058			
				ω0	0.11399	1.00000	0.11399	1.00000			
				ω1	0.11399	1.00000	19.63740	19.63740			
	Model A null	15	-9147.279352	1							Not Allowed
**Gya_TLR21_2**	Model A	28	-14048.172839	Site class	0	1	2a	2b	Model A vs.Model A null	0.000003564	25 D 0.952*, 427 D 0.973*, 513 Q 0.956*, 552 R 0.998**, 577 D 0.973*
				f	0.55406	0.39020	0.03270	0.02303			615 M 0.976*,653 R 0.963*,770 D 0.975*,809 V 0.972*,815 S 0.974*
				ω0	0.06857	1.00000	0.06857	1.00000			
				ω1	0.06857	1.00000	53.35043	53.35043			
	Model A null	27	-14058.915882	1							Not Allowed

a Only PSS with significant posterior probabilities are indicated. Asterisks indicate posterior probabilities P≥95%(*) and p>99%(**). LRT Statistics (2ΔL) and *p*-values for the branch-site models on the ancestral Nototheniidae branch for Tlr5, Tlr8, Tlr13, Tlr22, Tlr23, and *G. acuticeps* Tlr21 duplicate (Tlr21_2).

"w" symbol is Omega which is calculated by Codeml. This value indicates the classes of sites analyzed (emphasis on estimated).

PSS were also found within the TM and TIR domains of Tlr5 (**Supplementary Figure 12**), Tlr8 (**Supplementary Figure 13B**), Tlr21 (**Supplementary Figure 14B**), and Tlr22 (**Supplementary Figure 14C**). The teleost-specific receptor, Tlr22, was highly modified with 44 PSS identified (SM, *p* < 0.001), of which 41 were localized within the ectodomain and 27 within LRRs (**Figure 4** and **Tables 1**, **2**). The second most modified receptor in Nototheniidae was Tlr8 with 12 PSS (SM, *p* < 0.001) within the ectodomain and 3 within LRRs (**Figure 4A** and **Tables 1**, **2**). For *G. acuticeps* Tlr21 duplicates (Tlr21_2), 7 PSS were within the ectodomain, of which 3 mapped within LRR, suggesting functional divergence after species-specific gene duplication (**Figure 4** and **Tables 1**, **2**). PSS were also found for Nototheniidae Tlr13 [1 site within LRRs (SM, *p* = 0.004)] and Tlr23 [2 sites, of which one was within LRRs (SM, *p* = 0.0004)]. For Tlr5 and Tlr22, a single and two PSS were detected, respectively, within the TM region. PSS in the TIR domains were also detected for Tlr8 (1 PSS), Tlr21 (3 PSS), and Tlr22 (1 PSS).

**Table 2:** Selective pressure analysis for the site model.

Receptor	Model	np	Ln L	Estimates of parameters				Model compared	LRT P-value	Positive sites^a^
**TLR5**	M2a	14	-2969.998729	p:	0.62742	0.36347	0.00911	M1a vs. M2a	1	No significant sites found
				ω:	0.10344	1.00000	4.70001			
	M1a	12	-2970.783667	p:	0.63311	0.36689				Not Allowed
				ω:	0.10281	1.00000				
	M8	14	-2969.998943	p0=0.94815	p=0.38730	q=0.78363		M7 vs.M8	0.296470369	
				(p1= 0.05185)	ω= 1.98531					
	M7	12	-2971.214751	p=0.27144		q=0.44370				Not Allowed
										
										
**TLR8**	M2a	14	-5382.496766	p:	0.29522	0.65107	0.05371	M1a vs. M2a	0.000000000	No significant sites found
				ω:	0.00000	1.00000	13.82641			
	M1a	12	-5417.313267	p:	0.42715	0.57285				Not Allowed
				ω:	0.00000	1.00000				
	M8	14	-5388.003906	p0=0.74956	p=0.00500	q=1.72148		M7 vs.M8	0.000000000	25 G 0.999**,27 K 0.987*,28 E 0.988*,48 W 1.000**,63 Q 0.988*,92 S 0.987*,275 V 0.995**,414 S 0.984*,501 K 0.985*,753 H 0.985*,761 K 0.987*
				(p1= 0.25044)	ω= 5.39259					
	M7	12	-5418.056161	p=0.02508		q=0.01283				Not Allowed
										
										
**TLR13**	M2a	12	-3257.897218	p:	0.73321	0.00000	0.26679	M1a vs. M2a	0.008925285	No significant sites found
				ω:	0.00000	1.00000	2.89533			
	M1a	10	-3262.616085	p:	0.46579	0.53421				Not Allowed
				ω:	0.00000	1.00000				
	M8	12	-3257.897218	p0=0.73321	p=0.00500	q=2.09224		M7 vs.M8	0.003965279	210 H 0.963*
				(p1= 0.26679)	ω= 2.89538					
	M7	10	-3263.427397	p=0.02050		q=0.00751				Not Allowed
										
										
**TLR22**	M2a	16	-5046.424216	p:	0.61339	0.15062	0.23598	M1a vs. M2a	0.000000000	No significant sites found
				ω:	0.00000	1.00000	5.25653			
	M1a	14	-5086.643355	p:	0.48532	0.51468				Not Allowed
				ω:	0.00000	1.00000				
	M8	16	-5046.642792	p0=0.69561	p=0.00500	q=1.39574		M7 vs.M8	0.000000000	24 T 0.963*,32 R 0.968*,36 A 0.957*,40 G 0.957*,45 S 0.999**,49 P 0.964*,66 K 0.951*,88 T 0.999**,119 K 0.994**,141 R 0.994**,149 T 0.951*,181 R 0.969*
				(p1= 0.30439)	ω= 4.52587					245 E 0.993**,254 S 0.969*,257 T 0.995**,273 Q 0.981*,300 H 0.992**,302 R 0.983*,328 L 0.996**,347 R 0.994**,368 Y 0.958*,373 Q 0.962*,
										395 E 0.995**,397 Y 0.954*,419 A 0.954*,423 S 0.964*,447 N 0.994**,477 V 0.995**,496 Q 0.957*,498 P 0.962*,501 T 0.998**,503 R 0.957*
										520 S 0.968*,544 N 0.991**,559 N 0.991**,571 S 0.972*,590 R 0.950*,642 E 0.954*,663 P 0.969*,707 T 0.977*,709 S 1.000**,760 T 0.960*,
	M7	14	-5090.346350	p=0.03100		q=0.01510				Not Allowed
										
										
**TLR23**	M2a	12	-4560.594806	p:	0.79957	0.00000	0.20043	M1a vs. M2a	0.000457781	No significant sites found
				ω:	0.17464	1.00000	3.91573			
	M1a	10	-4568.283926	p:	0.38807	0.61193				Not Allowed
				ω:	0.00000	1.00000				
	M8	12	-4560.595092	p0=0.79997	p=5.98663	q=27.59025		M7 vs.M8	0.000389425	333 T 0.976*, 515 S 0.974*
	M7	10	-4568.445931	p=0.02094		q=0.00655	0.00655			Not Allowed

^a^Only PSS with significant posterior probabilities are indicated. Asterisks indicate posterior probabilities P≥95%(*) and p>99%(**). LRT Statistics (2ΔL) and *p*-values for the site models on the Nototheniidae sequences of Tlr5, Tlr8, Tlr13, Tlr22, and Tlr23.

"w" symbol is Omega which is calculated by Codeml. This value indicates the classes of sites analyzed (emphasis on estimated).

### 3.2 Tlr Response to Immune and Temperature Challenge in Nototheniidae *N. rossii*

LPS injection modified the activity of immune-related enzyme activity in the Nototheniidae *N. rossii* but *tlr* gene expression only responded to increased seawater temperature (**Supplementary Figure 15**). LPS injections provoked a change in *N. rossii* immune-related enzymes, and lysozyme activity in plasma was significantly higher (*p* = 0.012) after 24 h when compared to the control group at 2°C, suggestive of an immune response (**Supplementary Figure 15A**). However, no statistically significant differences in plasma antitrypsin activity or expression of *tlr5*, *tlr21*, and *tlr22* in the immune-related organs, the head–kidney and intestine, were detected between the control and LPS-challenged groups at 2°C (**Supplementary Figures 15B–G**). Comparison between animals maintained at 2°C and at 6°C revealed a significant increase (*n* = 6, *p* < 0.001) in *tlr5* gene expression in the head–kidney of control and LPS-challenged fish at 6°C. In the head–kidney of control and LPS-challenged fish after 8 h, *tlr21* (*n* = 6, *p* < 0.001) and *tlr22* (*n* = 6, *p* = 0.005) transcripts were significantly increased in the 6°C compared to the 2°C group. In the intestine, *tlr21* was significantly downregulated (*n* = 6, *p* < 0.05) after 24 h in both control and LPS-challenged groups at 6°C compared to the 2°C group (**Supplementary Figures 15C–G**).

## 4 Discussion

The Nototheniidae possess a similar *tlr* gene number and repertoire to other teleosts, suggesting that speciation and adaptation to the Antarctic environment had little impact on receptor gene number. The only exception was *D. mawsoni*, with only 10 Tlrs. The general protein structure of Tlrs in Nototheniidae was well conserved, although codon usage bias revealed significant modifications in the ectodomain and LRR motifs of Tlr5, Tlr8, Tlr13, Tlr22, and Tlr23. This suggests that subtle modifications in ligand binding characteristics evolved, presumably an adaptation to the unique microbial community found in the frigid Antarctic waters.

### 4.1 Adaptation to Antarctic Has Not Played a Significant Role in *tlr* Gene Repertoire

Comparative analysis of the genomes and transcriptome of Nototheniidae fishes revealed that the *tlr* gene repertoire was similar to other Perciformes. This suggests that their recent radiation in the cold-stable Antarctica environment was not associated with significant gain or loss of the *tlr* gene repertoire as described for other gene families that contributed to Nototheniidae adaptations such as the expansion of antifreeze glycoproteins and zona pellucida proteins that confer protection from ice damage (65–67). The exception was *D. mawsoni*, an early evolving lineage of the Nototheniidae where species-specific *tlr* gene deletions occurred.

Of the seven Nototheniidae genomes analyzed, *D. mawsoni* retained the lowest number of *tlr* genes and linkage analysis revealed that the homologues of *tlr1*, *tlr2* (*a* and *b*), *tlr5*, and *tlr8* were deleted from its genome. The reason for *tlr* gene deletion in *D. mawsoni* is unclear but may be associated with its unique physiological adaptations. This species is considered the best model for studies of the evolutionary underpinning of secondary pelagicism (absence of a swim-bladder) in the Antarctic clade. The neutral buoyancy of *D. mawsoni* arose from significant genetic reprograming to favor fat deposition and modified bone development (68). Morphological alterations in *D. mawsoni* to reduce body density and promote static lift include increased lipid deposits under skin and musculature and minimized bone mineralization (69) with an upregulation of genes involved in adipogenesis, triacylglycerol synthesis, and fat storage in skeletal muscle and by favoring chondrogenesis over osteoblastogenesis (68). In mammals, TLR non-immune functions have been linked to bone development and increased osteoclast differentiation (70), both of which are supressed in *D. mawsoni* (69), raising the possibility that in the Nototheniidae and teleosts in general, *tlr* gene evolution may not only be influenced by their role in innate immunity.

An unanticipated result from our expanded comparative analysis of the *tlr* gene repertoire in fish was the reduced *tlr* gene number identified in the Pleuronectiform order (flatfish). The number of *tlr* gene members found in the four species analyzed was variable and *S. senegalensis* and *C. semilaevis* had the most compact gene repertoire with 6 (*tlr1*, *tlr3*, *tlr5*, *tlr7*, *tlr8*, and *tlr22*) and 7 (*tlr14/18*, *tlr3*, *tlr5*, *tlr7*, *tlr8*, *tlr9*, and *tlr22*) *tlrs* genes, respectively. Like the Nototheniidae, extant flatfish of the Pleuronectiformes represent a monophyletic group (71). In the Nototheniidae and Pleuronectiform radiations, chromosomal rearrangements and chromosomal fusions occurred independently in different species lineages (35, 72). The compaction and reorganization of the genome in these two teleost lineages do not explain *tlr* gene deletions as the gene synteny was well conserved. An intriguing observation is the common physiological characteristic of *D. mawsoni* and adult flatfishes as both lack a swim bladder and the organisms have a high content of polyunsaturated fatty acids (73). In mammals changes in lipid metabolism and increased lipid storage affected innate immunity and caused modifications in Tlr signaling, endocytosis, and cytokine secretion (74, 75). In fact, dyslipidemia and other metabolic diseases in mammals are closely connected to an altered immune response (76, 77). Taking into consideration the interaction between lipid metabolism and Tlr activity, these morphological/metabolic adaptations may explain the reduced Tlr repertoire in flatfish and *D. mawsoni*.

Identification of specific gene duplicates in Notothenioid fish compared to other teleosts [e.g., zona pellucida (ZP)-like proteins (78), mitochondrial proteins (79), and antifreeze glycoprotein (65, 80)] has been associated with diversification of protein function as a consequence of their adaptation to the freezing environment (68). However, conservation of species-specific duplicates of TLR subfamilies occurred in the case of the *tlr21* gene in the *G. acuticeps* genome and *tlr2* in all Nototheniidae and other teleosts and it was missing from *D mawsoni*. Compared to previous reports of the *tlr* repertoire in *N. coriiceps*, our extended comparative analysis identified 3 additional *tlr* genes, *tlr2b*, *tlr13*, and *tlr25* (23). A similar *tlr* gene repertoire was retrieved from the transcriptome of the congeneric species, *N. rossii*, further confirming that *tlr* gene members are conserved across Nototheniidae. Comparison of Antarctic Nototheniidae with the sub-Antarctic Bovichtidae representative, *C. gobio*, revealed loss of *tlr5s* and *tlr23*, and in the closest Perciform *G. aculeatus* (Gasterosteidae family), *tlr2b* and *tlr23* were lost, indicating that *tlr* gene evolution was lineage and species-specific. The TLR11 subfamily is best conserved in aquatic vertebrates particularly the Nototheniidae. The TLR11 subfamily member, *tlr13*, was upregulated in the Perciform, *Miichthys miiuy*, infected with *Vibrio anguillarum*, a bacterium that causes hemorrhagic septicemia (81), and also by viral stimulation using poly(I:C) (82, 83). Studies in the future of fish Tlr–ligand interactions will be essential to decipher the link between the TLR repertoire, infection, innate immune activation, and disease resistance.

In the Nototheniidae and the sister lineage Bovichtidae, *tlr* gene number was similar to other teleosts while the Gadiformes are peculiar as exuberant lineage-specific duplications and deletion events restricted to a few *tlr* genes occurred. For example, in *G. morhua*, 7 copies for *tlr8*, 14 copies *tlr22*, and 9 copies for *tlr25* were found in the genome and *tlr5*/*tlr5*S, *tlr1*, and *tlr2* genes were missing, and this has been associated with the loss of *mhc II* and expansion of *mhc I* genes (32, 84). The large *tlr* gene expansion in *G. morhua* was correlated with the highly variable pathogen loads and community composition of the paleoclimatic Arctic conditions (32). However, we propose that the cod is an exception and its unique TLR repertoire and highly modified innate gene repertoire is a result of the number and frequency of repetitive tandem repeats within genes and gene promoter regions and significant genome rearrangement/recombination compared to other teleosts (85, 86).

### 4.2 Notothenioid Tlrs Evolved Under Positive Selection Pressures

The accepted consensus is that TLRs are functionally conserved (87). However, evidence from more refined analysis of the evolution of ligand recognition indicates positive selection of TLR3, TLR4, TLR5, and TLR15 in birds (88); TLR4 and TLR7 in wild rodents (89); and TLR1, TLR2, TLR6, and TLR8 (90) in pigs and cetacean TLRs (91). Evidence from the present study indicates that in Antarctic Nototheniidae, positive selection at the ectodomain, which recognize pathogen molecules, and within the LRR motifs of Tlr5, Tlr8, Tlr13, Tlr21, Tlr22, and Tlr23 occurred. This was presumably driven by the unique microbiome that has emerged in Antarctic due to the environment and species isolation caused by the circumpolar currents. Of note was the high representation of members of the TLR11 superfamily, a member-rich family in teleosts, although in Antarctic Nototheniidae, the TLR11 superfamily underwent a unique evolution compared to other vertebrates.

The results of the present analysis contrast with those of a previous study in *T. bernacchii* in which positive selection of Tlr2 was proposed to result from their radiation in an Antarctic environment (92). Based on our in-depth analysis of TLR evolution, we propose that although the general framework of the protein was conserved, the ligand interacting domain changed in Antarctic Nototheniidae as an adaptation to the unique microbiota. Based on the results, we propose that TLR evolution mirrors in some ways what occurs in antibodies where the antibody structure is highly conserved and changes in small protein segments (hypervariable regions) establish the exquisitely specific antigen recognition (93). Our TLR model explains the conundrum arising from the highly conserved TLR repertoire across most vertebrates despite their exposure to vastly different microbe loads, communities, and the surface or molecular characteristics of their pathogens.

The members of the TLR11 superfamily compared to other TLR families possess the largest number of LRRs and respond to viral infections (25, 94, 95). Protein modeling studies of Tlr22 LRRs in *D. rerio* revealed that changes in the LRR motif due to PSS modified ectodomain structure towards a flattened horseshoe-shape conformation and caused a functional change towards sensing long-sized dsRNA (96, 97). Positive selection in the *tlr22* duplicates of *G. morhua* and *Boleophthalmus pectinirostris* (32, 98–100) was also associated with species-specific pathogen recognition. PSS of the duplicate Tlr21_2 TIR domain in *G. acuticeps* falls within the three conserved regions, box 1(YDAFISY), box 2 (SSKLC-RD-PG), and box 3 (a conserved W surrounded by basic residues), which are involved in signal transduction (1 and 2) and receptor localization (3) (101). Previous studies in *Larimichthys polyactis* suggested that the TIR domain of Tlr21 mediates activation of the factor, nuclear kappa B (102), suggesting that divergent signaling may occur in the duplicate *tlr21* in *G. acuticeps*. PSS were also detected in Tlr8 LRRs (known to be efficient against virus) and in the TM domain of Tlr5 (that mostly react to bacteria), and changes in the homologue receptor sequences in other teleosts were associated with adaptation to different environmental pathogens (103). Functional data on amino acid mutations in the receptor TM domain is scarce but a TM mutation identified in Nototheniidae Tlr5 (PSS 530) is located near an important binding site in mammals for the plasma membrane auxiliary protein UNC93B1, which is responsible for receptor trafficking and localization (104), although the functional relevance of the Nototheniidae Tlr5 mutation is unclear as plasma membrane auxiliary proteins for Tlr have not yet been identified in teleosts.

### 4.3 Notothenioid Tlr Response to LPS Is Species-Specific and Changes With Temperature Increase

The distribution of the *tlr* transcripts in head–kidney (*tlr5*, *tlr21*, and *tlr22*) and intestine (*tlr21* and *tlr22*) of *N. rossii* confirmed their previously described tissue-specific distribution (23, 105). In LPS-stimulated *N. rossii* (8 h and 24 h later), no changes in *tlr* gene expression were detected, which conflicts with previous studies in other teleost fish where these genes were modified. For example, in *L. polyactis* head–kidney and spleen, *tlr21* and *tlr22* were differentially expressed 6–12 h post LPS challenge (100); in *C. carpio*, *tlr22* expression was increased in head–kidney (106); and in *Acipenser dabryanus*, *tlr21* and *tlr22* expression was increased in the head–kidney leukocytes (105). Moreover, in the head–kidney of *N. coriiceps*, *tlr5* and *tlr22* were upregulated 12 h post-challenge with heat-killed bacteria (*E. coli* O11:B4) but *tlr21* expression was unaltered (23). In *D. rerio* and *O. mykiss* head–kidney *tlr5* expression was also found to be responsive to bacteria flagella (24, 107–109).

The reason for the difference between the response to LPS of *N. rossii* (*E. coli* O111:B4) in the present study compared to *N. coriiceps* and other teleosts was unclear, particularly since the glycoprotein endotoxin, LPS, is a major component of the outer cell wall membrane of all Gram-negative bacteria and is a common bacterial PAMP. However, it should be noted that although bacterial LPS is considered to be identical between different bacteria and strains (110), recent studies revealed chemical divergence between Gram-negative species (111, 112) and within bacterial species with different phenotypes (112, 113). This may partially explain the lack of response to LPS in our study; in fact, cold adapted Gram-negative bacteria have a modified cell wall including the structure of LPS within the O-antigen region (110). The stimulation of lysozyme activity 24 h after LPS challenge in *N. rossii* is similar to what has been previously described in *S. salar* (114, 115). The lysozymes are a diverse and poorly conserved group of non-specific innate immune enzymes that hydrolyze the peptidoglycan layer of the bacterial cell wall and several genes were recently reported in Notothenioids (116). The functional requirement of peptidoglycan residues for lysozyme activity (117) and the detected response of *N. rossii* in our study highlight that the LPS challenge activated an innate immune response. The absence of a *tlr* response to this typical bacterial PAMP, although puzzling, may be attributed to a range of factors including LRR region specificity, the characteristics of the LPS (mammalian bacterial origin), or an inappropriate route, dose, or duration of exposure.

Differences in the responsiveness of *N. rossii* and *N. coriiceps* to an LPS challenge were previously reported in relation to iron metabolism genes (118) with *N. coriiceps* being more responsive. In the present study, increased seawater temperature had a more profound effect than LPS on *tlr5*, *tlr21*, and *tlr22* gene expression in *N. rossii*. The effect of temperature on the regulation of *tlr* expression has previously been reported in *G. morhua* where an increase from 4 to 12°C increased expression of *tlr21* and *tlr22* in the head–kidney and spleen (98), although in *D. rerio*, temperature stress (from 23°C to 31°C) did not significantly modify *tlr21* and *tlr22* expression in the head–kidney and spleen (97). Interestingly, in mammals, heat shock proteins (HSP) 60 and 70 are known to be mediators of TLR2 and TLR4 expression (119). In teleosts that are ectotherms and for which there is a rich literature about the importance of the HSP in response to a thermal challenge, their potential involvement in TLR signaling is not studied (120, 121). An interesting nuance in the case of Antarctic Notothenioids is the loss of HSP function in those that have been studied, which raises questions that remain to be resolved about the potential mediators of the TLR response to LPS under thermal challenge in *N. rossii* (122–124).

## 5 Conclusion

**Figure 5:**
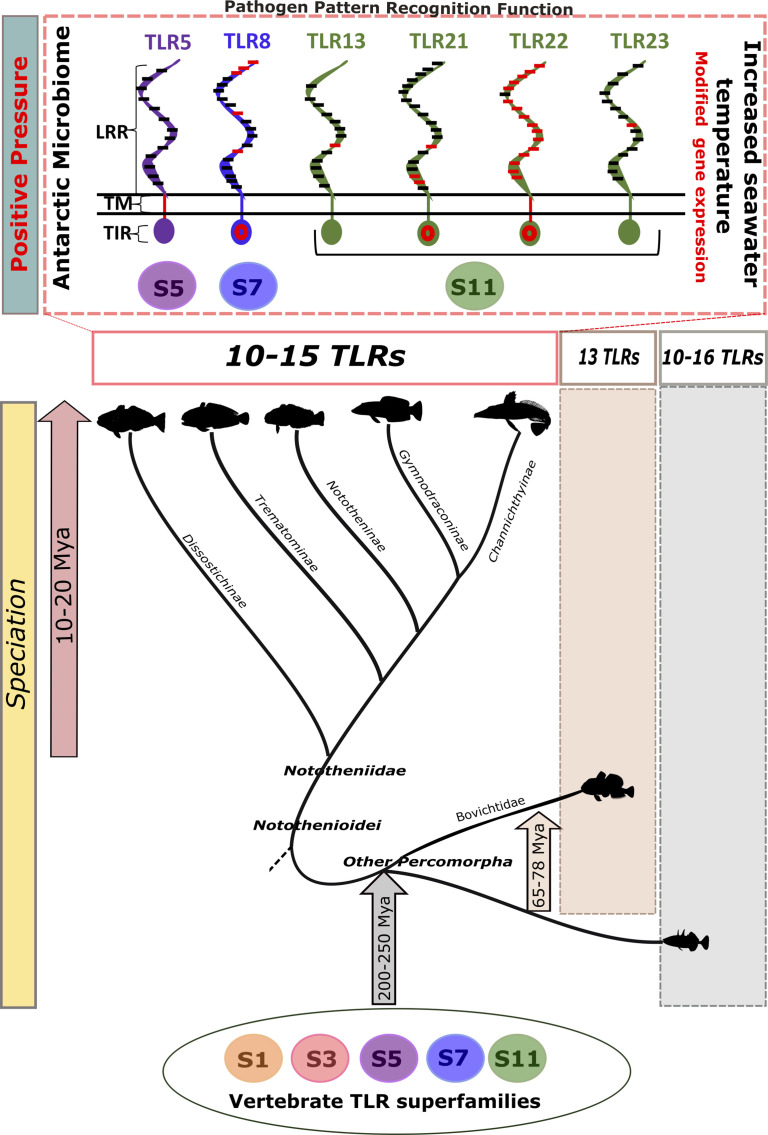
Explaining TLR evolution in Nototheniidae. The Antarctic fish speciation and associated unique adaptations to extreme cold occurred 10–20 million years ago (Mya) but did not result in profound modifications of the TLR gene complement. Representatives of the five vertebrate TLR superfamilies exist in Nototheniids, and gene number and evolutionary origin were common with those in other teleosts and vertebrates. Notable changes in TLRs (Tlr5, Tlr8, Tlr13, Tlr21, Tlr22, and Tlr23) arose from positive evolutionary pressure acting on the LRR motifs that determine receptor function and pattern recognition due presumably to the unique Antarctic microbiome. *Tlr* expression was temperature sensitive and indicates that pathogen detection may be modified under future climate change scenarios. The five TLR superfamilies (S) are represented by numbers and with colored circles. The positive selective pressure sites are highlighted on the receptor conceptual structure with red lines and red circles. LRR: leucine-rich repeat, TM: transmembrane, and TIR: Toll-interleukin 1 receptor. Speciation times for fish lineages were obtained from (35). The scheme is not drawn to scale.

Our study provides a comprehensive description and comparative analysis of the evolution of the TLR system in Nototheniidae. We reveal that apart from *D. mawsoni*, no major gene family expansion or contraction occurred in Nototheniidae, and the *tlr* gene complement was conserved across most teleosts. In contrast, within the LRR motif of the ectodomain, which recognize pathogen molecules, positive selection was detected in Tlr5, Tlr8, Tlr13, Tlr22, and Tlr23 across all the analyzed Antarctic Notothenioids. This was presumably driven by the unique environment and microbiome in the Antarctic and suggests that functional divergence in pathogen recognition may exist. We noted parallels in the pattern of evolution of *tlr*s characterized by gene deletions in *D. mawsoni* and Pleuronectiformes, which may be explained by the extreme changes in lipid metabolism and a rearranged skeleton that resulted from adaptation to a pelagic (68) or benthic lifestyle. In *N. rossii*, although LPS stimulation modified plasma lysozyme activity, it failed to change the expression of *tlr5*, *tlr21*, and *tlr22* expressed in the head–kidney and intestine, which were targeted in our analyses. In contrast, an increase in water temperature (2°C to 6°C) caused a significant increase in *tlr* expression, although the mediators of this response remain to be established. These results indicate that increased temperatures associated with climate change are likely to directly modify the immune response mediated by TLRs. In summary, our study demonstrated that the rapid speciation and adaptation to freezing water temperatures did not play an important role in the evolution of Tlr number in Nototheniidae but was associated with a shift in the LRR pathogen recognition domain that was common across all the Nototheniidae analyzed, and of the six subfamilies of TLR in Nototheniidae fishes, Tlr22 was the most modified (**Figure 5**).

## Data Availability Statement

The datasets presented in this study can be found in online repositories. The names of the repository/repositories and accession number(s) can be found in the article/**Supplementary Material**.

## Ethics Statement

Animal collection and experimentation were approved by the Portuguese Environment Agency, under the regulations set by the Treaty of Madrid for scientific investigation in Antarctica. The experiments performed complied with EU and Portuguese regulations for animal experimentation.

## Author Contributions

DP, JC, CS, and AC conceived and designed the study. CS and PG carried out the animal experiments and collected the samples. CS and WZ performed the lab experiments. CS, JC, YW, and SF performed bioinformatic analysis. CS, AC, JC, LC, and DP contributed to data analysis and interpretation. CS, SF, JC, and DP drafted the manuscript. All authors contributed to the article and approved the submitted version.

## Funding

This study was funded by the Portuguese Foundation for Science and Technology (FCT) through projects PTDC/BIAANM/3484/2014, Natural Science Foundation of China (No. 41761134050), Foundation of Science and Technology Commission of Shanghai (No. 19590750500), and FCT-NSFC/0002/2016 and UIDB/04326/2020, FACC PROPOLAR (2016/2017). CS received a fellowship SFRH/BD/120040/2016.

## Conflict of Interest

The authors declare that the research was conducted in the absence of any commercial or financial relationships that could be construed as a potential conflict of interest.

## Publisher’s Note

All claims expressed in this article are solely those of the authors and do not necessarily represent those of their affiliated organizations, or those of the publisher, the editors and the reviewers. Any product that may be evaluated in this article, or claim that may be made by its manufacturer, is not guaranteed or endorsed by the publisher.
