# Supplementary material for: Toll-Like Receptor Evolution: Does Temperature Matter?
Source: Front Immunol. 2022 Feb 14;13:812890. doi: 10.3389/fimmu.2022.812890 (PMC8882821; doi:10.3389/fimmu.2022.812890)

Supplementary Material

1. Supplementary Methods
2. Supplementary Results
3. References
4. Supplementary Figure legends
5. Supplementary Tables
6. Supplementary Figures

**1 Supplementary Methods**

The detailed methods performed for the selective pressure analysis performed are described below. The amino acid positions given in the text refer to the positions in the edited alignment that was used in the analysis.

- 1. **Branch-Site analysis**

The Branch-Site model was used to identify positive selection acting on a few sites of a predefined branch (foreground branch) by allowing *ω* to vary both across the branches and the sites of the phylogeny (Zhang *et al,* 2005). The alternative model A assumes four classes of sites where site class 0 presents 0 < ω0 < 1 for both background and foreground branches, class 1 with *ω*1 > 1 for both background and foreground branches, class 2a with 0 < ω0 < 1 for the background branches and *ω*2 > 1 for the foreground branches and class 2b where *ω*1 = 1 for the background branches and *ω*2 > 1 for the for foreground branches (Zhang et al., 2005). This model is then compared to an identical null model but where *ω*2 = 1 fixed (Zhang et al., 2005).

- 1. **Sites analysis**

Two pairs of models M1a (nearly neutral) vs M2a (positive selection) and M7 (beta) vs M8 (beta& *ω*) are useful for the identification of positively selected sites Wong *et al.,* (2004). Both consist in the comparison of a model where *ω* in all sites are limited to 0 < *ω* ≤ 1 (M1a, M7) with a model which allows for *ω* > 1 (M2a, M8). The models M1a and M2a allow for two classes of sites. M1a where ω0 is estimated from the data and may vary between 0 < ω0 < 1 and ω1 = 1 which is fixed. M2a allows for one more sites class ω2, where ω2 > 1 (Wong *et al.,* 2004). The beta model (M7) fixes a ratio on a distribution *ω* ≤ 1 in all lineages and implements ten classes of sites against the beta-*ω* model (M8) which also implements a fixed *ω* for all lineages but attributes 11 classes of sites, 10 sites with *ω* ≤ 1, and one site with *ω* > 1 to allow for positive selection (Wong *et al.,* 2004).

- 1. **Likelihood-Ratio tests**

The LRT analysis were conducted to compare a null model that does not allow for *ω* > 1 in the distribution with an alternative model that does (Yang *et al.,* 2005). For each model pairs the LRT statistic 2Δl, (twice the log likelihood difference) was compared with critical values retrieved from the χ^2^ distribution (significance level of 5%). In specific cases of sites and branch-site models where LRTs suggested positive selection, Bayes empirical Bayes (BEB) was used to calculate the posterior probabilities that each codon belongs to the site class of positive selection under their respective models (M2a, M8 for site models or alternative model A for branch-site model) (Yang, 2007). Sites were considered to be under positive selection if their posterior probability of *ω* > 1 was equal or higher than 0.95 (Nozawa, Suzuki, & Nei, 2009).

**2 Supplementary Results for selective pressure analysis**

For the Branch-site analysis the ancestral branch of Notothenioidei as well as the ancestral branch of Pleuronectiformes were tested whenever possible. From the 12 tlrs for which the ancestral branch of Notothenioidei was tested only two identified positive selection, Tlr3 with a significant p-value of 0.000102623 and Tlr22 with a significant p-value of 0.013081816 and Tlr3 presented 2 PSS (422 and 591) with posterior probability > 0.95 (Supplementary File 1). From the 5 tlrs for which the ancestral branch of Pleuronectiformes was tested none presented evidence of positive selection.

**3 References**

Ahn, D. H., Kang, S., and Park, H. Transcriptome analysis of immune response genes induced by pathogen agonists in the Antarctic bullhead notothen *Notothenia coriiceps*. Fish Shellfish Immunol (2016) 55: 315–322. doi:10.1016/j.fsi.2016.06.004.

Bai, S. C., and Lee, S. "Culture of Olive Flounder: Korean Perspective". In: Daniels, H. V. and Watanabe, W. O., editors. Practical Flatfish Culture Stock Enhancement, Blackwell (2010). p. 156–168. doi:10.1002/9780813810997.ch9.

Campos, C., Castanheira, M. F., Engrola, S., Valente, L. M. P., Fernandes, J. M. O., and Conceição, L. E. C. Rearing temperature affects Senegalese sole (*Solea senegalensis*) larvae protein metabolic capacity. Fish Physiol Biochem (2013) 39: 1485-1496. doi:10.1007/s10695-013-9802-x.

de Castro, E., Sigrist, C. J. A., Gattiker, A., Bulliard, V., Langendijk-Genevaux, P. S., Gasteiger, E., et al. ScanProsite: Detection of PROSITE signature matches and ProRule-associated functional and structural residues in proteins. Nucleic Acids Res (2006) 34: 362–5. doi:10.1093/nar/gkl124.

Fang, J., Tian, X., and Dong, S. The influence of water temperature and ration on the growth, body composition and energy budget of tongue sole (*Cynoglossus semilaevis*). Aquaculture (2010) 299: 106-114. doi:10.1016/j.aquaculture.2009.11.026.

Hughes, L. C., Ortí, G., Huang, Y., Sun, Y., Baldwin, C. C., Thompson, A. W., et al. Comprehensive phylogeny of ray-finned fishes (Actinopterygii) based on transcriptomic and genomic data. Proc Natl Acad Sci U S A (2018) 115: 6249 LP – 6254. doi:10.1073/pnas.1719358115.

Letunic, I., Khedkar, S., and Bork, P. SMART: Recent updates, new developments and status in 2020. Nucleic Acids Res (2021) 49: D458–D460. doi:10.1093/nar/gkaa937.

Liu, G., Zhang, H., Zhao, C., and Zhang, H. Evolutionary History of the Toll-Like Receptor Gene Family across Vertebrates. Genome Biol Evol (2019) 12: 3615–3634. doi:10.1093/gbe/evz266.

Mayden, R. L., Page, L. M., and Burr, B. M. A Field Guide to Freshwater Fishes of North America North of Mexico. Copeia (1992) 1992: 920. doi:10.2307/1446175.

Near, T. J., Dornburg, A., Harrington, R. C., Oliveira, C., Pietsch, T. W., Thacker, C. E., et al. Identification of the notothenioid sister lineage illuminates the biogeographic history of an Antarctic adaptive radiation. BMC Evol Biol (2015) 15: 109. doi:10.1186/s12862-015-0362-9.

Neilson, J. D., Waiwood, K. G., and Smith, S. J. Survival of Atlantic halibut (*Hippoglossus hippoglossus*) caught by longline and otter trawl gear. Can J Fish Aquat Sci (1989) 46: 887-897. doi:10.1139/f89-114.

Nielsen, H. "Predicting secretory proteins with signaIP". In: Kihara, D., editor. Protein Fuction Prediction: Methods and Protocols, Methods in Molecular Biology, Springer (2017) 611. p. 59–73. doi:10.1007/978- 1-4939-7015-5_6.

Nozawa, M., Suzuki, Y., and Nei, M. Reliabilities of identifying positive selection by the branchsite and the site-prediction methods. Proc Natl Acad Sci U S A (2009) 106: 6700–6705. doi:10.1073/pnas.0901855106.

Palti, Y. Toll-like receptors in bony fish: From genomics to function. Dev Comp Immunol (2011) 35: 1263–1272. doi:10.1016/j.dci.2011.03.006.

Wong, W. S. W., Yang, Z., Goldman, N., and Nielsen, R. Accuracy and power of statistical methods for detecting adaptive evolution in protein coding sequences and for identifying positively selected sites. Genetics (2004) 168: 1041–1051. doi:10.1534/genetics.104.031153.

Yang, Z. PAML 4: phylogenetic analysis by maximum likelihood. Mol Biol Evol (2007) 24: 1586– 1591. doi:10.1093/molbev/msm088.

Yang, Z., Wong, W. S. W., and Nielsen, R. Bayes empirical Bayes inference of amino acid sites under positive selection. Mol Biol Evol (2005) 22: 1107–1118. doi:10.1093/molbev/msi097.

Zhang, J., Nielsen, R., and Yang, Z. Evaluation of an improved branch-site likelihood method for detecting positive selection at the molecular level. Mol Biol Evol (2005) 22: 2472–2479. doi:10.1093/molbev/msi237.

# Supplementary Figures and Tables

## Supplementary Figures


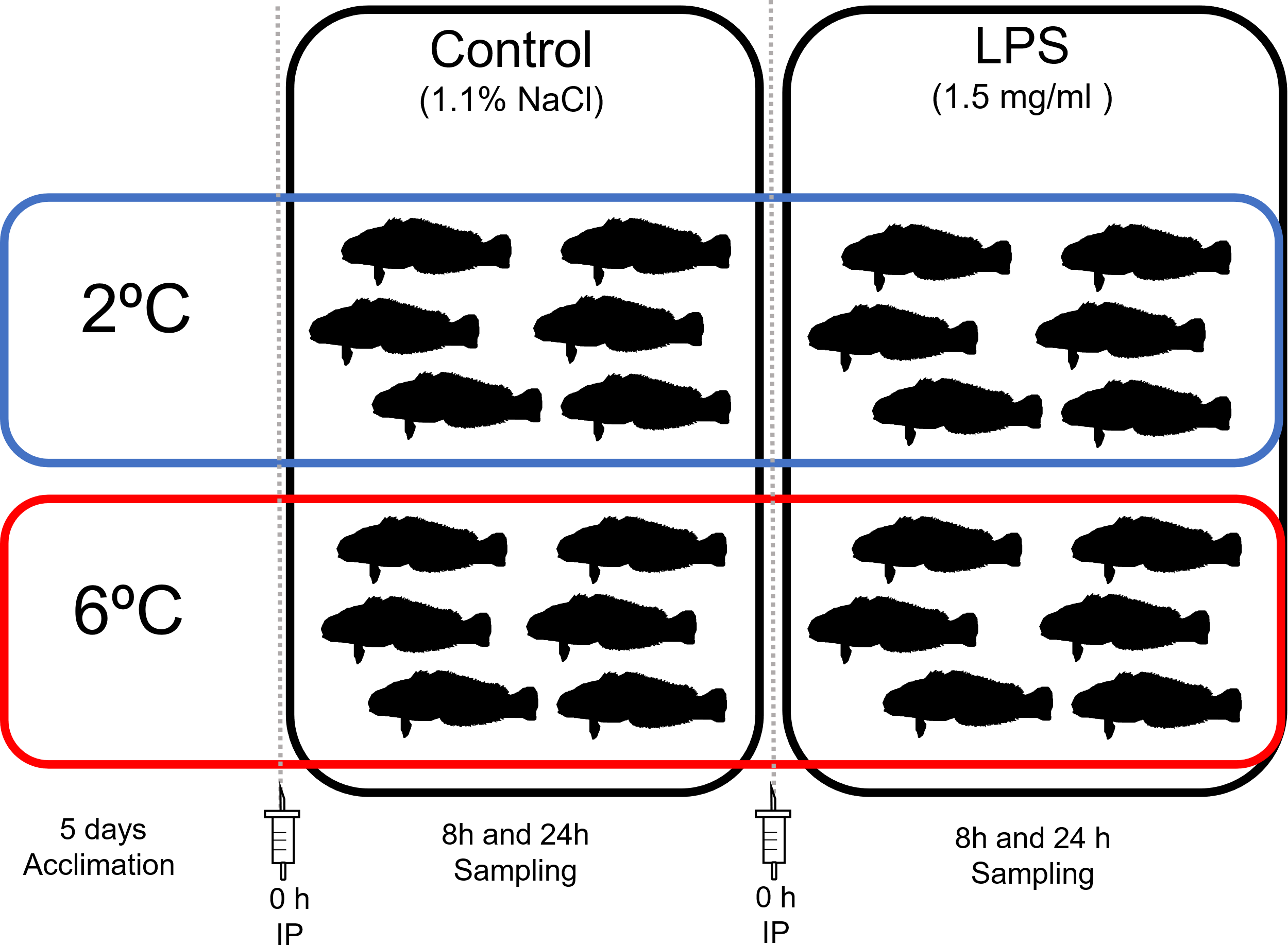


**Supplementary Figure 1. Schematic diagram of the experimental immune challenge.** Adult *N. rossii* captured from the wild were acclimated to the experimental circuit at 2 ºC (average seawater temperature) or an increased temperature of 6ºC before the immune challenge. For immune challenge groups of fish (n=6/ group) were exposed to bacterial lipopolysaccharides (LPS, 1.5 mg/ml) by I.P. injection at normal (2 ºC) or an increased water (6ºC) temperature. Control fish (sham) at both water temperatures were injected I.P. with saline buffer (0.2% (v/w) of 1.1% NaCl). Tissue samples and plasma were collected at 8 h and 24 h post-LPS challenge.


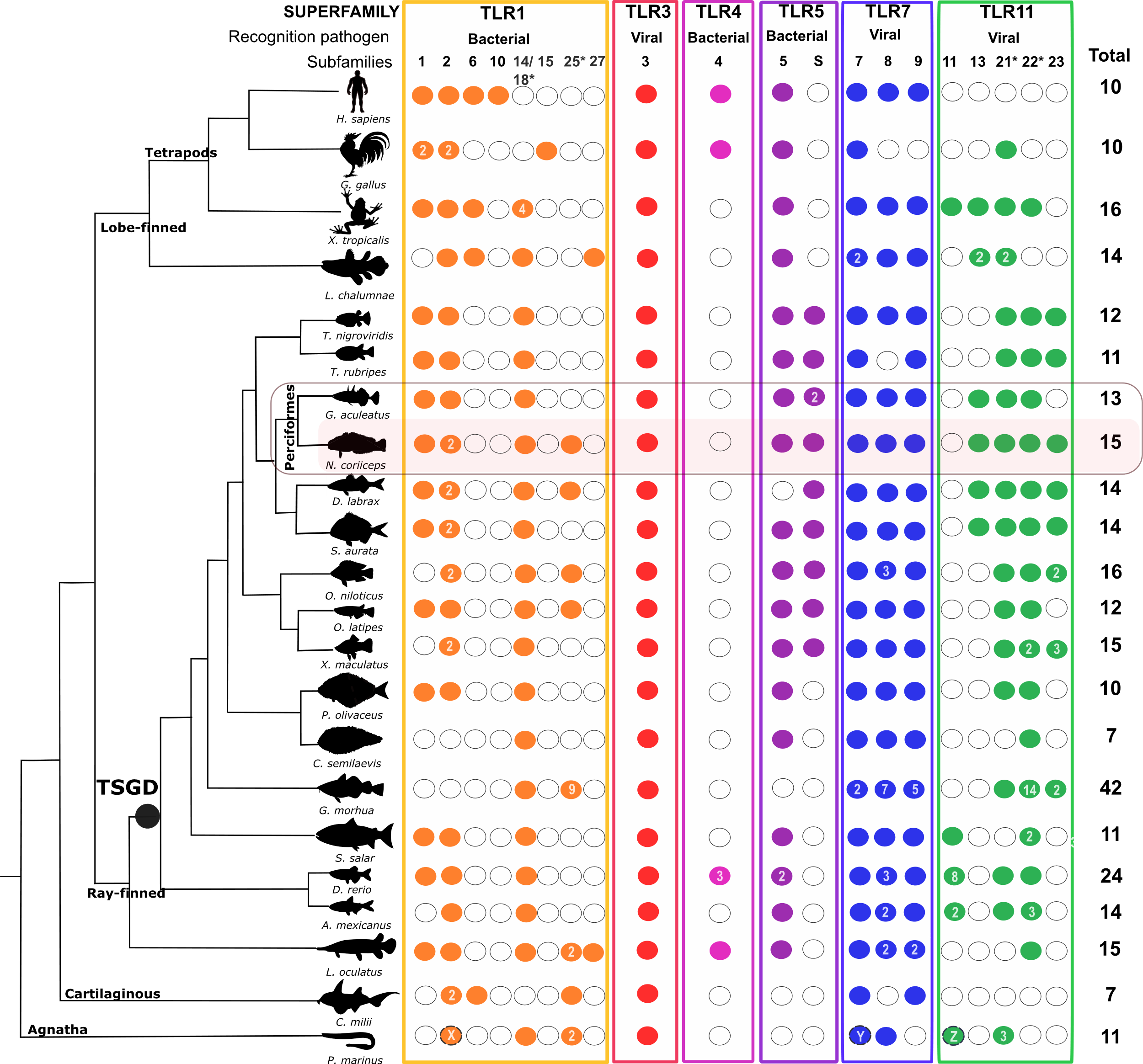


**Supplementary Figure 2.** **Dendrogram illustrating the *TLRs* found in vertebrates.** The genes identified belonging to the six vertebrate TLR superfamilies are represented by coloured circles, when multiple genes were identified gene number is indicated inside the circle. Gene absence is indicated by a white circle. The profile of pathogen recognition for each TLR superfamily is indicated (Liu, Zhang, Zhao, & Zhang, 2019). The subfamily members that recognize a wider range of viruses and bacteria are indicated by “*” (Liu et al., 2019; Palti, 2011). *N. coriiceps* is included as a representative of the Notothenioidei fish. The Perciform branch to which the Notothenioidei belong is circled and the Eupercaria clade annotated to highlight the Antarctic representatives with the phylogenetically proximate teleosts (Hughes et al., 2018). TLR superfamily members from human, chicken, reptile and Xenopus genomes are also represented. The total number of genes found is indicated for each species. The figure was constructed taking into consideration the evolutionary relationship between the species using as the starting point the studies of (Ahn, Kang, & Park, 2016; Near et al., 2015). The teleost specific genome duplication (TSGD) event is indicated. Gene/transcript accession numbers are available in Supplementary Table 1.


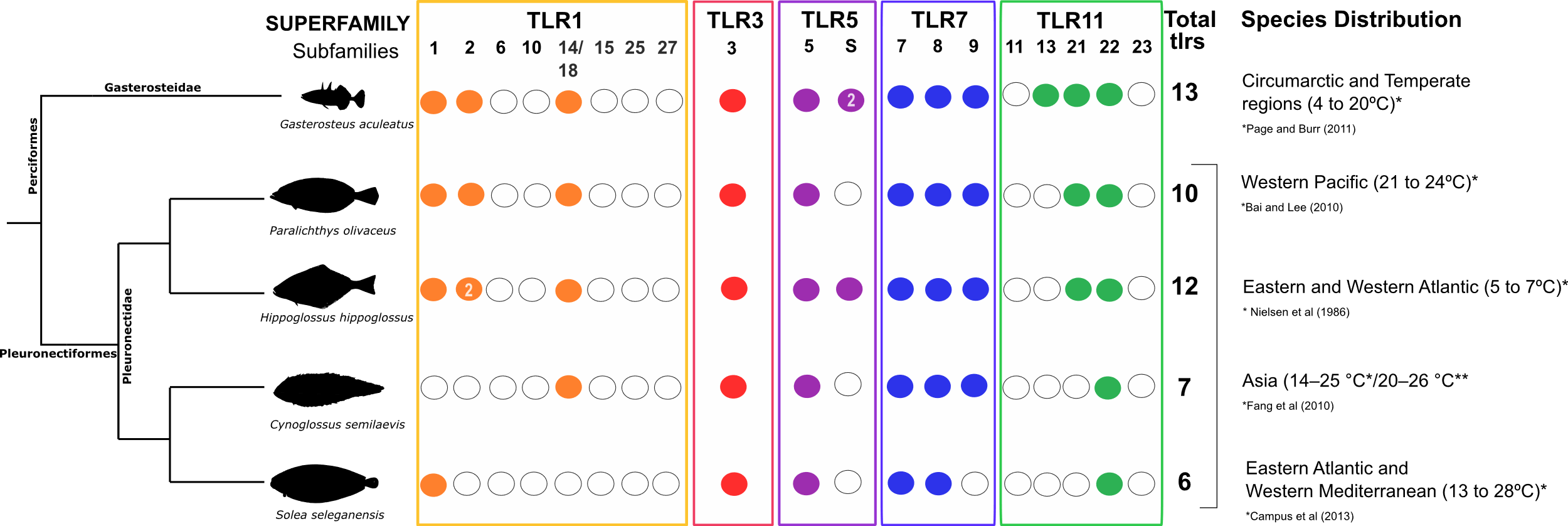


**Supplementary Figure 3. Detailed dendrogram of the tlrs members identified in Pleuronectiformes. The** genes identified are represented within the six vertebrate TLR superfamily by coloured circles, when multiple genes were identified gene number is indicated inside the circles. Gene absence is indicated by a white circle. The *G. aculeatus* was included as a representative of the Perciformes for comparisons with the Pleuronectidae order and considering species distribution (Bai & Lee, 2010; Campos et al., 2013; Fang, Tian, & Dong, 2010; Mayden, Page, & Burr, 1992; Neilson, Waiwood, & Smith, 1989). Genes were obtained by searching their genome assemblies. Accession numbers are available as Supplementary Table 1.

**Supplementary Figure 4. Complete BI phylogenetic tree of the Nototheniidae and other fish and tetrapod TLRs.** Branches of the six vertebrate TLR superfamilies are identified by the corresponding numbers within black circles and with different colours: S1 indicates TLR1 superfamily (orange); S3 for TLR3 superfamily (red); S4 for TLR4 superfamily (pink); S5 for TLR5 superfamily; S7 for TLR7 superfamily (blue) and S11 for TLR11 superfamily (green). Within each superfamily cluster when multiple subfamilies exist, they were coloured with variations of the main representative colour and are also indicated. Accession numbers of the sequences used to construct the tree are available in Supplementary Table 1. The teleost *tlr2* duplicates were named as *tlr2a* and *tlr2b* and TLR15 were not used as they are only found in chicken and lizard. Tree was rooted with the Cnidarian Tlrs clade (Liu et al., 2019). The sea lamprey tlrs within the vertebrate TLR1, TLR7 and TLR11 superfamilies were named *tlrX*, *tlrY* and *tlrZ*, respectively since their identity assignment for the Tlr subfamilies was not clear. To facilitate their identification the *tlrs* from seven Antarctic species are highlighted in blue, other Perciformes are indicated by a black dot and Pleuronectiformes by a black star. This figure is available in pdf file.

**Supplementary Figures 5. ML phylogenetic tree of the Nototheniidae and other fish and tetrapod TLRs.** Branches of the six vertebrate TLR superfamilies are identified by the corresponding numbers within black circles and with different colours: S1 indicates TLR1 superfamily (orange); S3 for TLR3 superfamily (red); S4 for TLR4 superfamily (pink); S5 for TLR5 superfamily; S7 for TLR7 superfamily (blue) and S11 for TLR11 superfamily (green). Within each superfamily cluster when multiple subfamilies exist, they were coloured with variations of the main representative colour and are also indicated. Accession numbers of the sequences used to construct the tree are available in Supplementary Table 1. The teleost *tlr2* duplicates were named as *tlr2a* and *tlr2b* and TLR15 were not used as they are only found in chicken and lizard. Tree was rooted with the Cnidarian Tlrs clade (Liu et al., 2019). The sea lamprey tlrs within the vertebrate TLR1, TLR7 and TLR11 superfamilies were named *tlrX*, *tlrY* and *tlrZ*, respectively since their identity assignment for the Tlr subfamilies was not clear. To facilitate their identification the *tlrs* from seven Antarctic species are highlighted in blue, other Perciformes are indicated by a black dot and Pleuronectiformes by a black star. This figure is available in pdf file.


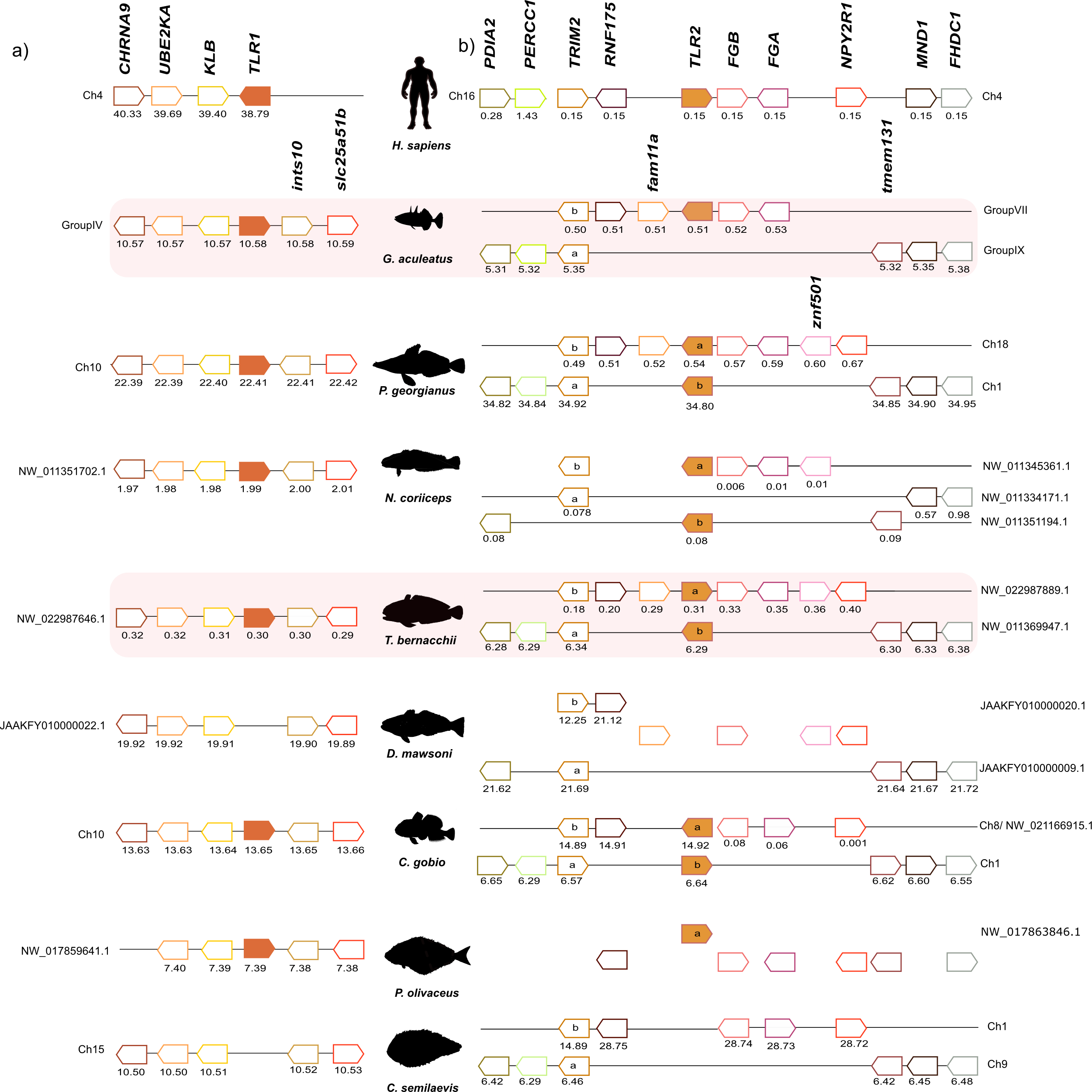


**Supplementary Figure 6. Gene synteny analysis of *tlr1* and *tlr2* in Antarctic Nototheniidae and other vertebrates**. A) represents the neighbouring gene environments of *tlr1* and B) *tlr2* in Antarctic Nototheniidae species. Presented are the Antarctic Nototheniidae *P. georgianus*, *N. coriiceps*, *T. bernacchii* and *D. mawsoni*, the representative of the sister lineage, *C. gobio*, *G. aculeatus* as a representative of the Perciform order, representatives of the Pleuronectiformes order, *P. olivaceus* and *C. semilaevis* and the tetrapod, *H. sapiens*. The gene environment of the *G. acuticeps* and *T. bernacchii* are assembled in chromosomes and were used as the reference. The genome regions analysed are indicated by a line and predicted genes are represented by arrows and the arrowheads indicate gene orientation in the genome; the gene symbol is given. The *tlr* genes are represented by fully coloured arrows: *tlr1* is in dark orange (A) and *tlr2* is in light orange (B). Duplicate genes are designated *a* and *b*. Neighbouring genes are represented by arrows outlined in different colours and the position of gene homologues is aligned. The gene positions in the genome assemblies analysed (Mega base pairs, Mbp) are indicated below each synteny map. Only common genes are represented. The positions of the *tlr2a* neighbouring genes found in the *P. olivaceus* genome (NW_017863846.1) are not indicated because they were found in different genome regions. The neighbouring genes represented are: cholinergic receptor nicotinic alpha 9 subunit (*chrn9a*), ubiquitin conjugating enzyme E2 K (*ube2ka*), klotho beta (*klb*), integrator complex subunit 10 (*ints10*), solute carrier family 25 member 51 (*slc25a51b*), protein disulphide isomerase family A member 2 (*pdia2*), proline and glutamate rich with coiled coil 1 (*percc1*), tripartite motif containing 2 (*trim2*), ring finger protein 175 (*rnf175*), family with sequence similarity 11 member A (*fam11a*), fibrinogen beta chain (*fgb*), fibrinogen alpha chain (*fga*), zinc finger protein 501 (*znf501*), transmembrane protein 131 (*tmem131*), meiotic nuclear division 1 (*mnd1*), FH2 domain containing 1 (*fhdc1*).


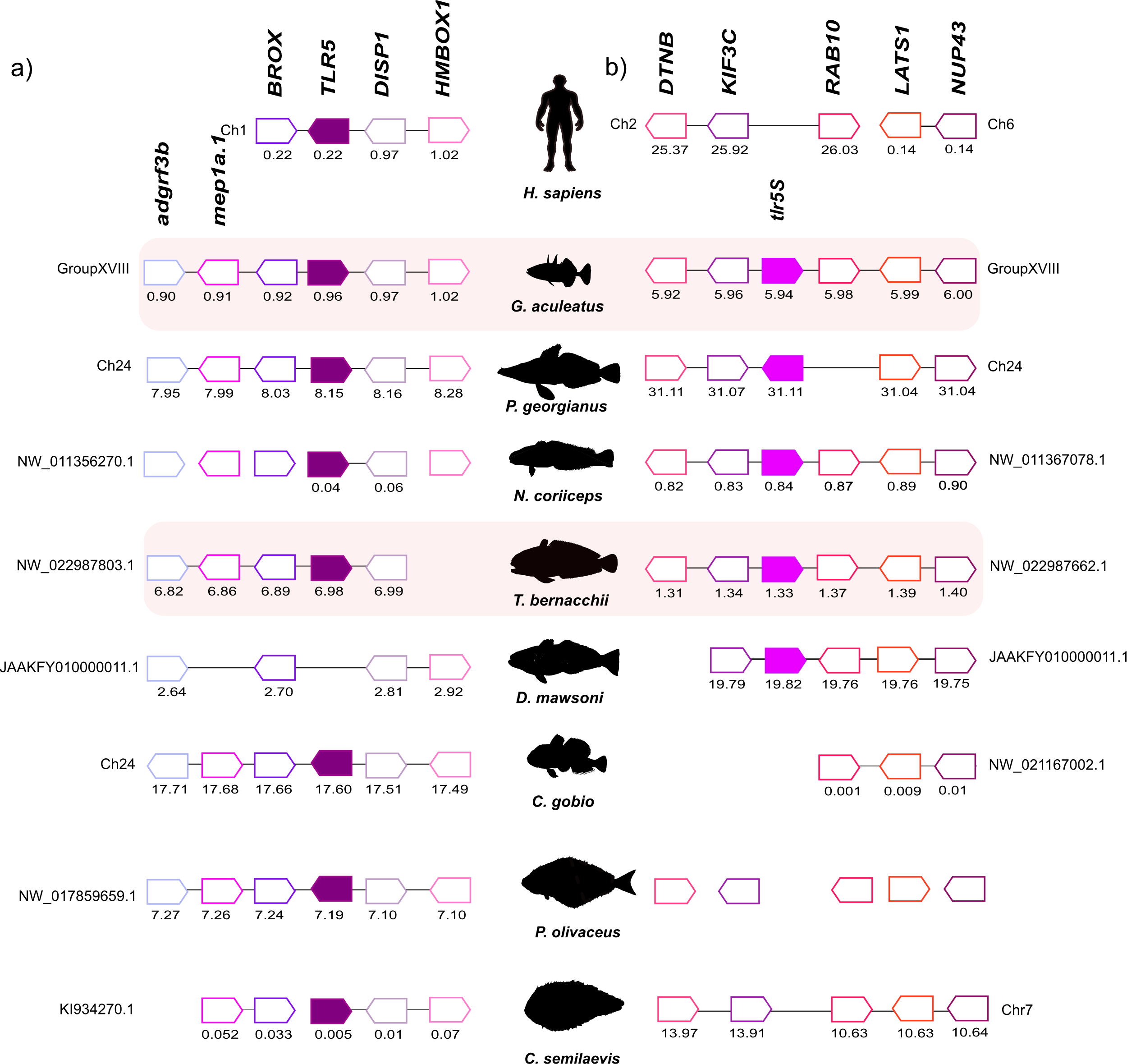


**Supplementary Figure 7. Gene synteny analysis of *tlr5* and *tlr5S* in Antarctic Nototheniidae and other vertebrates.** A) represents the neighbouring gene environments of *tlr5* and B) the neighbouring gene environments *of tlr5S*. Presented are the Antarctic Nototheniidae *P. georgianus*, *N. coriiceps*, *T. bernachii* and *D. mawsoni*. Other vertebrates include *C. gobio* as the representative of the sister lineage, *G. aculeatus* as a non-Antarctic representative of the Perciform order, two representatives of the Pleuronectiformes order, *P. olivaceus* and *C. semilaevis* and *H. sapiens* as the tetrapod. The gene environment of *tlr* genes in *G. acuticeps* and *T. bernacchii* for which the genome is assembled in chromosomes were used as the reference. In *G. aculeatus* (group XVII) and *P. georgianus* (chromosome 24) both genes map to the same genome regions. Genome regions analysed are indicated by a line and predicted genes are represented by arrows and the arrowhead indicates gene orientation and the gene symbol is given. *Tlr* genes are represented by fully coloured arrows: *tlr5* is dark purple (A) and *tlr5S* is light purple (B). Neighbouring gene families are represented by arrows outlined in different colours and the position of the gene homologues is aligned. Gene positions in the genome assemblies analysed (Mega base pairs, Mbp) are indicated below. Only common genes are represented. The positions of the *tlr5S* neighbouring gene homologues in the *P. olivaceus* genome (where *tlr5S* is absent) are not indicated because they were found in different genome regions. In the *G. aculeatus* genome the complete *tlr5S* gene is represented. Neighbouring genes represented are: adhesion G protein-coupled receptor F3b (*adgr3b*), meprin A alpha (PABA peptide hydrolase) tandem duplicate 1 (*mep1a.1*), BRO1 domain and CAAX motif containing (*brox*), dispatched RND transporter family member 1 (*disp1*), homeobox containing 1 (*hmbox1*), kinesin family member 13B (*kif13b*), dystrobrevin beta (*dtnb*), kinesin family member 3C (*kif3c*), ras-related protein Rab-10 (*rab10*), large tumour suppressor kinase 1 (*lats1*), nucleoporin 43 (*nup43*).


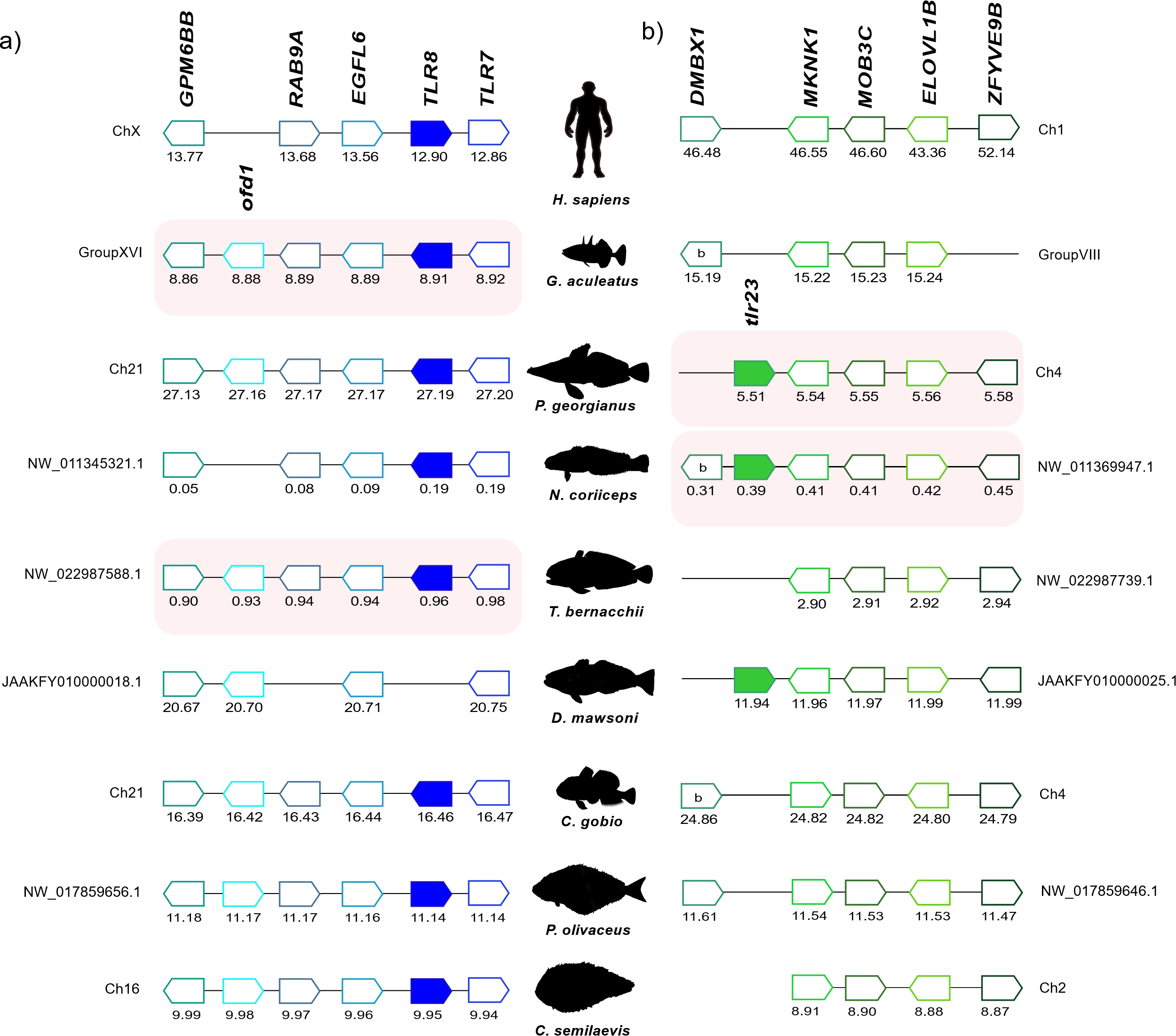


**Supplementary Figure 8. Gene synteny analysis of *tlr8* and *tlr23* in Antarctic Nototheniidae and other vertebrates.** A) represents the neighbouring gene environments of *tlr8* and B) the genes flanking *tlr23*. Antarctic Nototheniidae species represented are *P. georgianus*, *N. coriiceps*, *T. bernachii* and *D. mawsoni.* Other vertebrates include *C. gobio* as the representative of the sister lineage, *G. aculeatus* as a non-Antarctic representative of the Perciform order, two representatives of the Pleuronectiformes order, *P. olivaceus* and *C. semilaevis* and *H. sapiens* as the tetrapod. *G. aculeatus* and *T. bernacchii* that have their genome assembled in chromosomes were used as the reference for synteny maps of *tlr8*. For *tlr23* *P. georgianus* and *N. coriiceps* were used as the reference. The genome regions analysed are indicated by a line and predicted genes are represented by fully coloured arrows and the arrowhead indicates gene orientation in the genome and the gene symbol is given. *Tlr* genes are represented by coloured arrows: *tlr8* in blue (A) and *tlr23* in green (B). Neighbouring gene families are represented by arrows outlined in different colours and the position of the gene homologues is aligned. Only common genes are represented. Neighbouring genes represented are: glycoprotein M6BB (*gpm6bb*), oral-facial-digital syndrome 1 protein (*ofd1*), ras-related protein Rab-9A (*rab9a*), epidermal growth factor-like protein 6 (*egfl6*), toll-like receptor 7 (*tlr7*), diencephalon/mesencephalon homeobox 1 (*dmbx1*), MAP kinase-interacting serine/threonine-protein kinase 1 (*mknk1*), MOB kinase activator 3C (*mob3c*), elongation of very long chain fatty acids protein 1 (*elovl1b*), zinc finger FYVE-type containing 9B (*zfyve9b*).


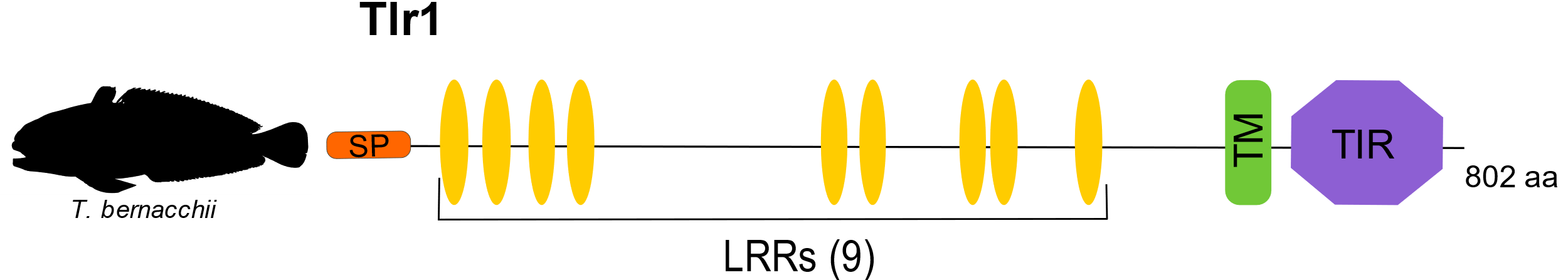


|  |  | **SP** | **LRR** | **TM** | **TIR** | **Size (aa)** |
| --- | --- | --- | --- | --- | --- | --- |
|  | *C. hamatus* | P | 9 | 1 | 1 | 800 |
| **Nototheniidae** | *P. georgianus* | P | 9 | 1 | 1 | 718 |
|  | *G. acuticeps* | P | 9 | 1 | 1 | 718 |
|  | *N. coriiceps* | P | 8 | 1 | 1 | 687* |
|  | *N. rossii* | P | 9 | 1 | 1 | 718 |
|  | *C. gobio* | ni | 9 | 1 | 1 | 717 |
|  | *G. aculeatus* | P | 9 | 1 | 1 | 717 |


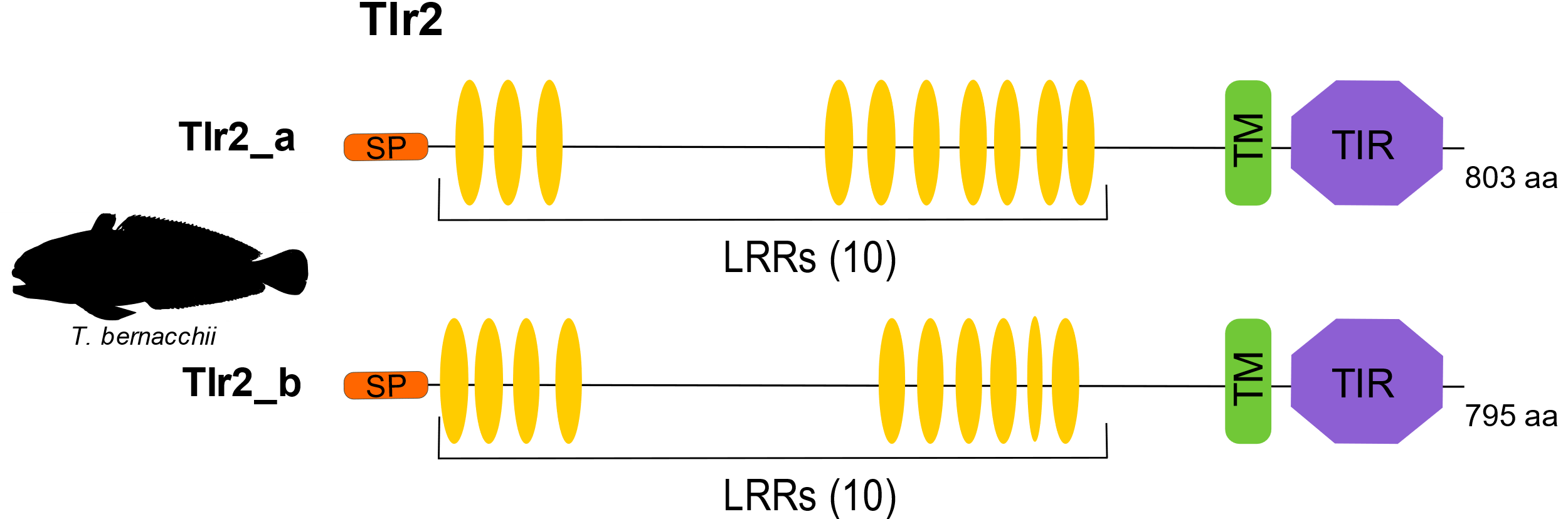


|  |  | **SP** | **LRR** | **TM** | **TIR** | **Size (aa)** |
| --- | --- | --- | --- | --- | --- | --- |
|  | *C. hamatus* | P | 11 | 1 | 1 | 802 |
|  |  | P | 10 | 1 | 1 | 804 |
| **Nototheniidae** | *P. georgianus* | P | 8 | 1 | 1 | 716 |
|  |  | P | 10 | 1 | 1 | 708 |
|  | *G. acuticeps* | P | 10 | 1 | 1 | 715 |
|  |  | P | 11 | 1 | 1 | 634* |
|  | *N. coriiceps* | ni | 2 | 1 | 1 | 263* |
|  |  | P | 10 | 1 | 1 | 714 |
|  | *N. rossii* | ni | 7 | 1 | 1 | 672* |
|  |  | ni | ni | ni | ni | 145* |
|  | *C. gobio* | ni | 12 | 1 | 1 | 778 |
|  |  | ni | 9 | 1 | 1 | 708 |
|  | *G. aculeatus* | ni | 8 | 1 | 1 | 718 |


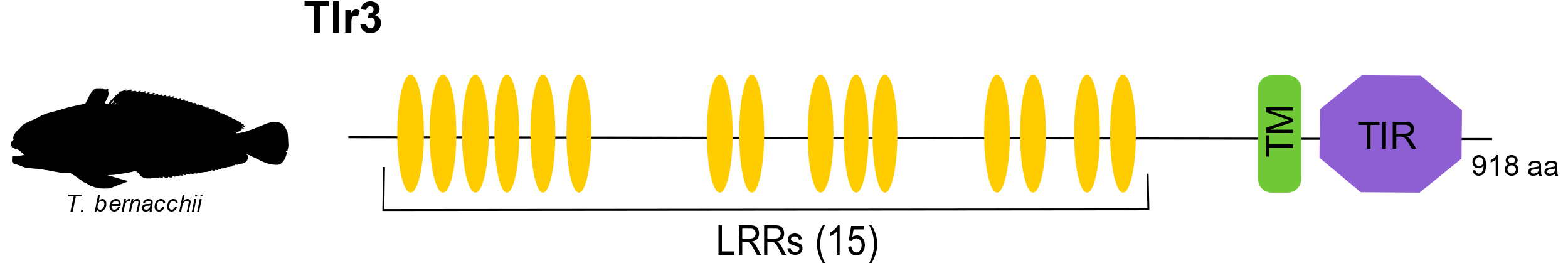


|  |  | **SP** | **LRR** | **TM** | **TIR** | **Size (aa)** |
| --- | --- | --- | --- | --- | --- | --- |
|  | *C. hamatus* | P | 17 | 1 | 1 | 842 |
| **Nototheniidae** | *P. georgianus* | ni | 17 | 1 | 1 | 803 |
|  | *G. acuticeps* | ni | 15 | 1 | 1 | 807 |
|  | *N. coriiceps* | ni | 17 | 1 | 1 | 810 |
|  | *N. rossii* | ni | 17 | 1 | 1 | 813 |
|  | *D. mawsoni* | ni | 6 | ni | ni | 305* |
|  | *C. gobio* | ni | 17 | 1 | 1 | 813 |
|  | *G. aculeatus* | ni | 14 | 1 | 1 | 814 |


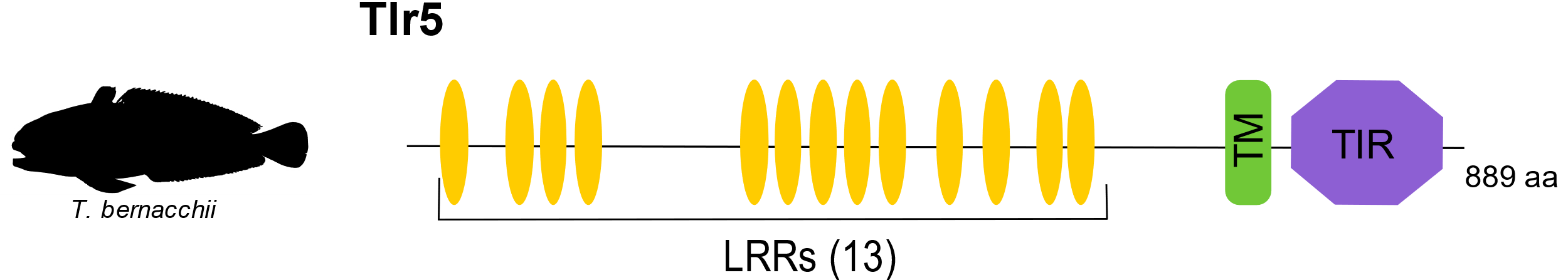


|  |  | **SP** | **LRR** | **TM** | **TIR** | **Size (aa)** |
| --- | --- | --- | --- | --- | --- | --- |
|  | *C. hamatus* | ni | 11 | 1 | 1 | 835 |
| **Nototheniidae** | *P. georgianus* | P | 12 | 1 | 1 | 895 |
|  | *G. acuticeps* | P | 12 | 1 | 1 | 889 |
|  | *N. coriiceps* | ni | 10 | 1 | 1 | 889 |
|  | *N. rossii* | ni | 8 | 1 | 1 | 587* |
|  | *C. gobio* | P | 10 | 1 | 1 | 889 |
|  | *G. aculeatus* | P | 12 | 1 | 1 | 883 |


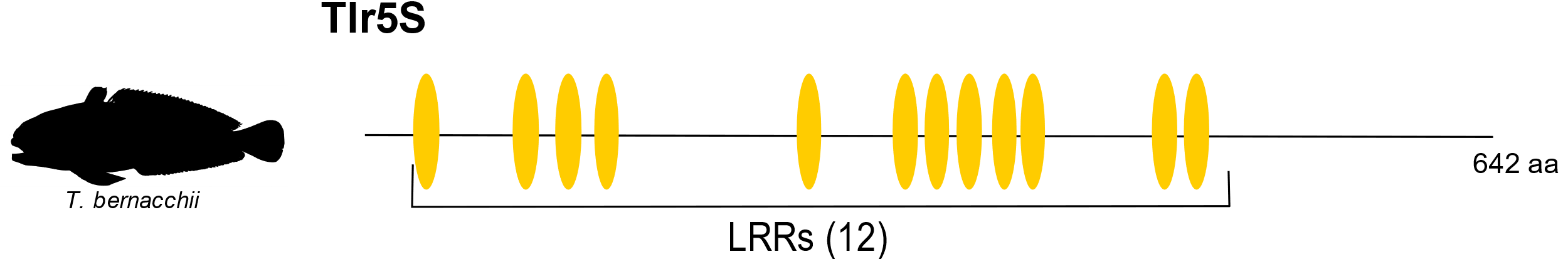


|  |  | **SP** | **LRR** | **TM** | **TIR** | **Size (aa)** |
| --- | --- | --- | --- | --- | --- | --- |
|  | *C. hamatus* | ni | 13 | ni | ni | 642 |
| **Nototheniidae** | *P. georgianus* | ni | 13 | ni | ni | 642 |
|  | *G. acuticeps* | ni | 12 | ni | ni | 642 |
|  | *N. coriiceps* | P | 12 | ni | ni | 642 |
|  | *D. mawsoni* | ni | 12 | ni | ni | 621 |
|  | *G. aculeatus* | P | 11 | ni | ni | 647 |
|  |  | ni | 6 | ni | ni | 265* |


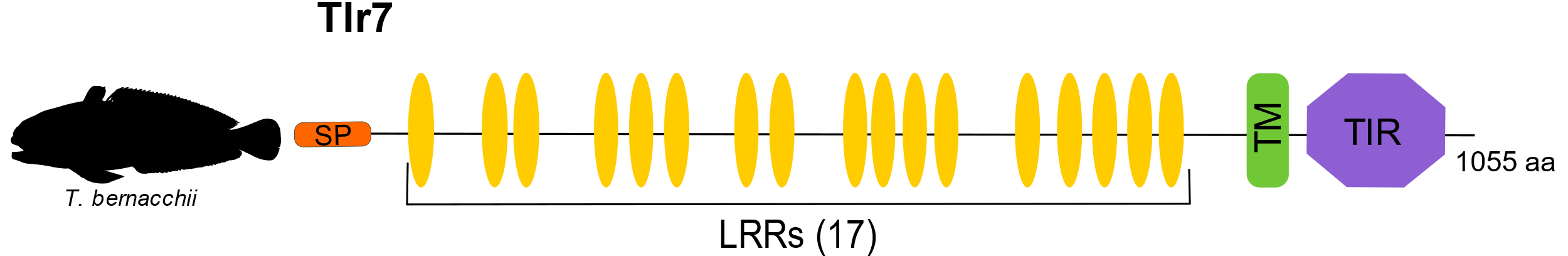


|  |  | **SP** | **LRR** | **TM** | **TIR** | **Size (aa)** |
| --- | --- | --- | --- | --- | --- | --- |
|  | *C. hamatus* | ni | 17 | 1 | 1 | 964 |
| **Nototheniidae** | *P. georgianus* | P | 17 | 1 | 1 | 946 |
|  | *G. acuticeps* | P | 17 | 1 | 1 | 946 |
|  | *N. coriiceps* | P | 17 | 1 | 1 | 946 |
|  | *N. rossii* | ni | 5 | ni | ni | 206* |
|  | *D. mawsoni* | P | 10 | 1 | 1 | 727* |
|  | *C. gobio* | P | 17 | 1 | 1 | 946 |
|  | *G. aculeatus* | P | 19 | 1 | 1 | 927 |


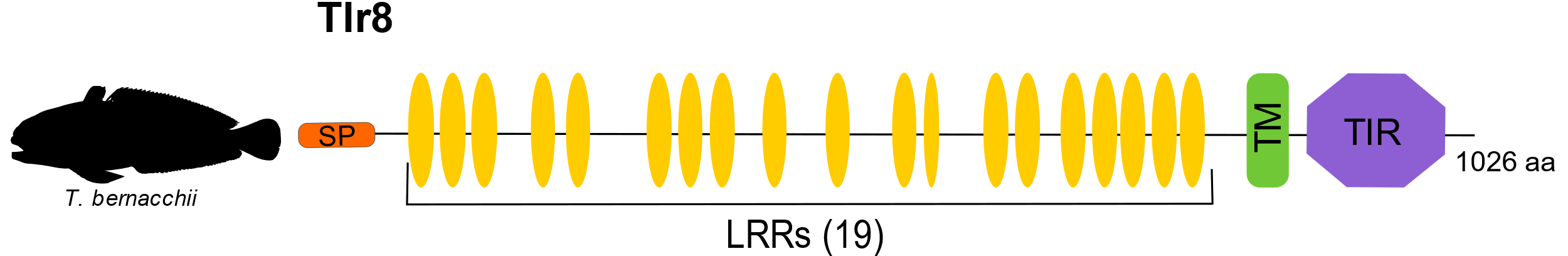


|  |  | **SP** | **LRR** | **TM** | **TIR** | **Size (aa)** |
| --- | --- | --- | --- | --- | --- | --- |
|  | *C. hamatus* | P | 18 | 1 | 1 | 1027 |
| **Nototheniidae** | *P. georgianus* | P | 18 | 1 | 1 | 913 |
|  | *G. acuticeps* | P | 18 | 1 | 1 | 913 |
|  | *N. coriiceps* | P | 18 | 1 | 1 | 913 |
|  | *N. rossii* | P | 18 | 1 | 1 | 913 |
|  | *C. gobio* | P | 19 | 1 | 1 | 911 |
|  | *G. aculeatus* | ni | 18 | 1 | 1 | 912 |


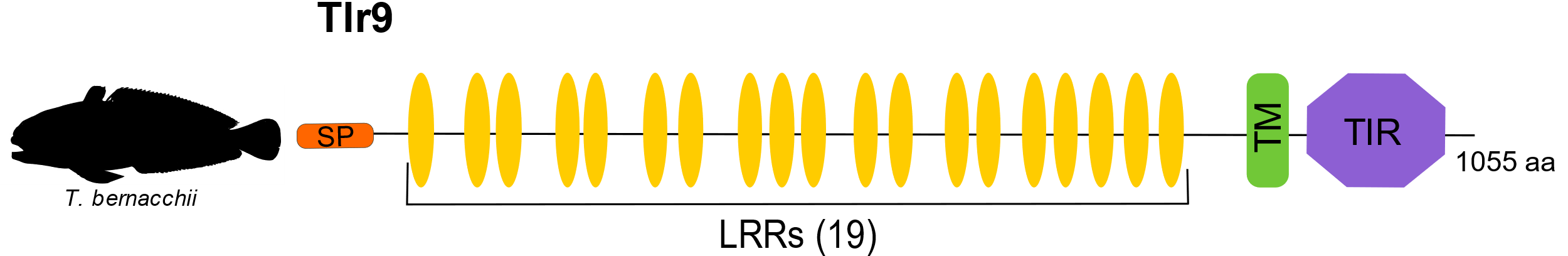


|  |  | **SP** | **LRR** | **TM** | **TIR** | **Size (aa)** |
| --- | --- | --- | --- | --- | --- | --- |
|  | *C. hamatus* | P | 20 | 1 | 1 | 939 |
| **Nototheniidae** | *P. georgianus* | P | 20 | 1 | 1 | 939 |
|  | *G. acuticeps* | P | 19 | 1 | 1 | 939 |
|  | *N. coriiceps* | P | 19 | 1 | 1 | 941 |
|  | *N. rossii* | P | 18 | 1 | 1 | 941 |
|  | *D. mawsoni* | P | 19 | 1 | ni | 865* |
|  | *C. gobio* | P | 18 | 1 | 1 | 939 |
|  | *G. aculeatus* | P | 16 | 1 | 1 | 939 |


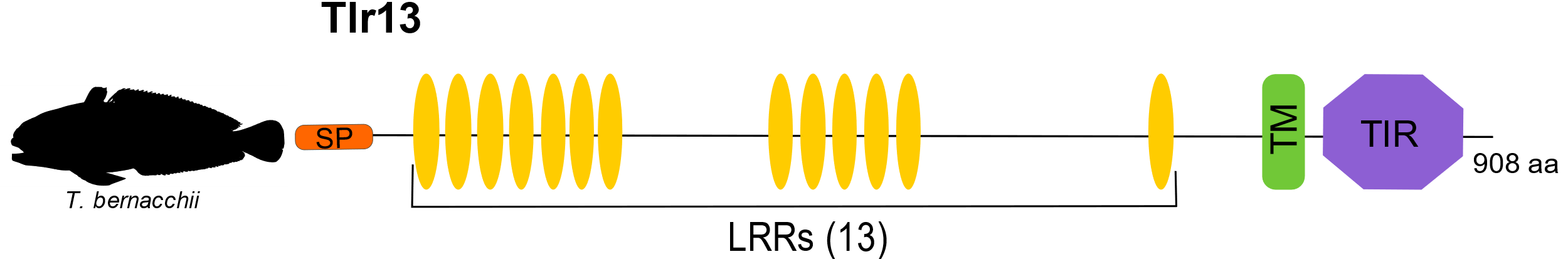


|  |  | **SP** | **LRR** | **TM** | **TIR** | **Size (aa)** |
| --- | --- | --- | --- | --- | --- | --- |
|  | *C. hamatus* | ni | ni | 1 | 1 | 150* |
| **Nototheniidae** | *P. georgianus* | P | 15 | 1 | 1 | 820 |
|  | *G. acuticeps* | P | 15 | 1 | 1 | 820 |
|  | *N. coriiceps* | ni | 11 | 1 | 1 | 996 |
|  | *D. mawsoni* | ni | 10 | 1 | 1 | 698* |
|  | *C. gobio* | P | 11 | 1 | 1 | 827 |
|  | *G. aculeatus* | ni | 10 | 1 | 1 | 816 |


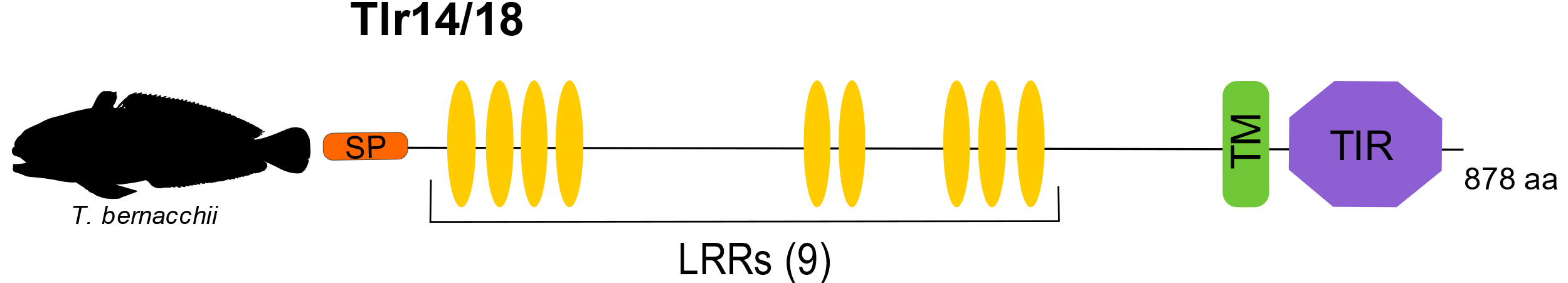


|  |  | **SP** | **LRR** | **TM** | **TIR** | **Size (aa)** |
| --- | --- | --- | --- | --- | --- | --- |
|  | *C. hamatus* | P | 9 | 1 | 1 | 878 |
| **Nototheniidae** | *P. georgianus* | P | 9 | 1 | 1 | 722 |
|  | *G. acuticeps* | P | 9 | 1 | 1 | 722 |
|  | *N. coriiceps* | P | 9 | 1 | 1 | 722 |
|  | *N. rossii* | P | 9 | 1 | 1 | 722 |
|  | *D. mawsoni* | ni | 5 | 1 | 1 | 454* |
|  | *C. gobio* | P | 8 | 1 | 1 | 722 |
|  | *G. aculeatus* | ni | 10 | 1 | 1 | 724 |


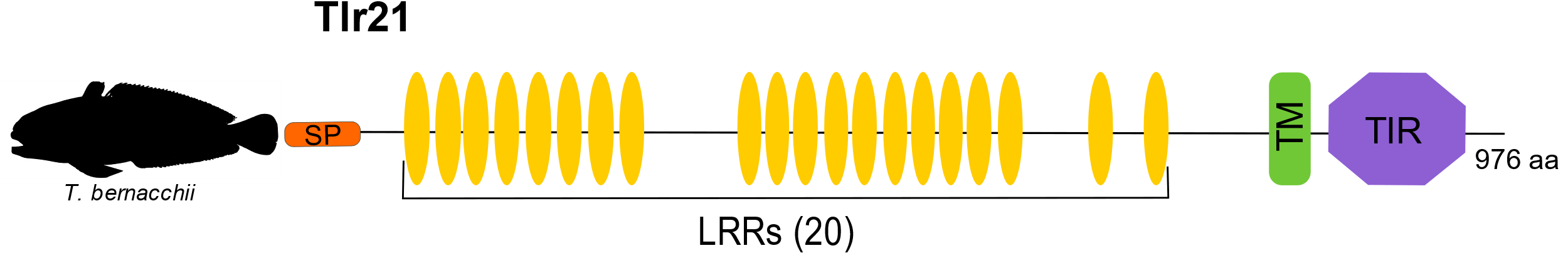


|  |  | **SP** | **LRR** | **TM** | **TIR** | **Size (aa)** |
| --- | --- | --- | --- | --- | --- | --- |
|  | *C. hamatus* | P | 18 | 1 | 1 | 978 |
| **Nototheniidae** | *P. georgianus* | P | 18 | 1 | 1 | 978 |
|  | *G. acuticeps* | P | 18 | 1 | 1 | 976 |
|  | *N. coriiceps* | P | 19 | 1 | 1 | 973 |
|  | *N. rossii* | ni | 17 | 1 | 1 | 996 |
|  | *D. mawsoni* | ni | ni | ni | 1 | 197* |
|  | *C. gobio* | P | 17 | 1 | 1 | 974 |
|  | *G. aculeatus* | ni | 15 | 1 | 1 | 928 |


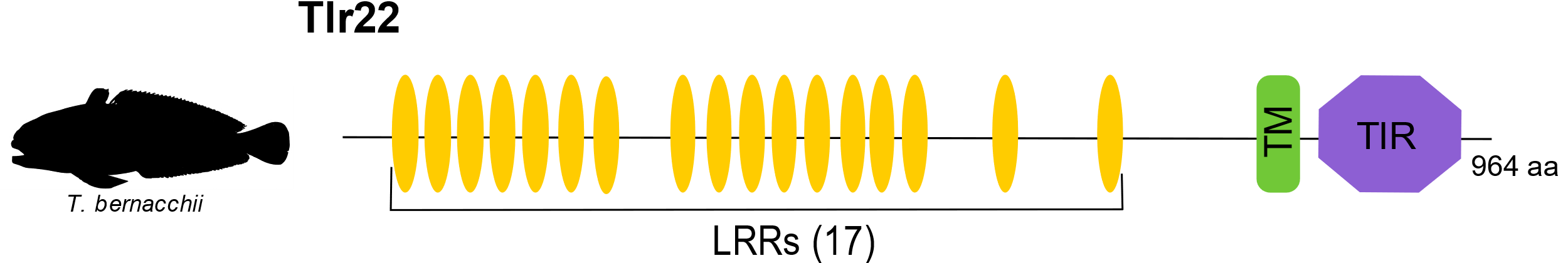


|  |  | **SP** | **LRR** | **TM** | **TIR** | **Size (aa)** |
| --- | --- | --- | --- | --- | --- | --- |
|  | *C. hamatus* | ni | 17 | 1 | 1 | 856 |
| **Nototheniidae** | *P. georgianus* | ni | 17 | 1 | 1 | 962 |
|  | *G. acuticeps* | ni | 17 | 1 | 1 | 962 |
|  | *N. coriiceps* | ni | 17 | 1 | 1 | 819 |
|  | *N. rossii* | ni | 16 | 1 | 1 | 1022 |
|  | *D.mawsoni* | P | 18 | 1 | 1 | 960 |
|  | *C. gobio* | ni | 17 | 1 | 1 | 942 |
|  | *G. aculeatus* | ni | 18 | 1 | 1 | 961 |


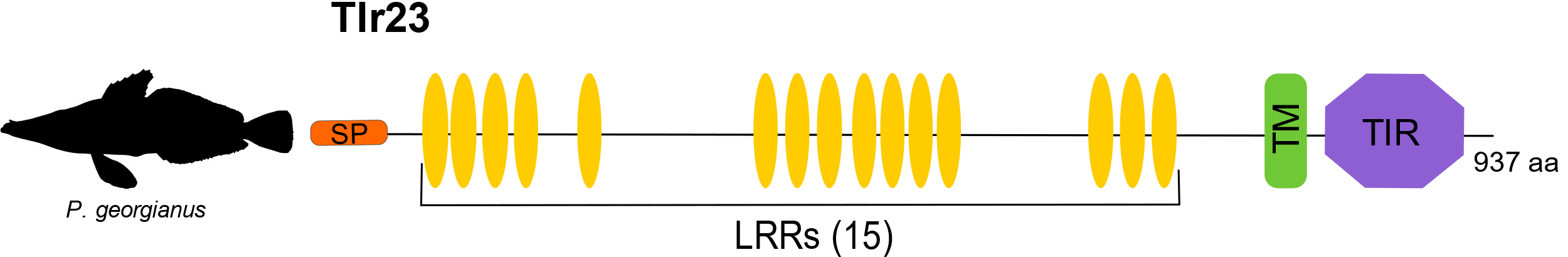


|  |  | **SP** | **LRR** | **TM** | **TIR** | **Size (aa)** |
| --- | --- | --- | --- | --- | --- | --- |
|  | *C. hamatus* | P | 15 | 1 | 1 | 948 |
| **Nototheniidae** | *G. acuticeps* | P | 15 | 1 | 1 | 824 |
|  | *N. coriiceps* | P | 13 | 1 | 1 | 799 |
|  | *N. rossii* | ni | 9 | 1 | 1 | 625* |
|  | *D. mawsoni* | P | 14 | 1 | 1 | 798 |


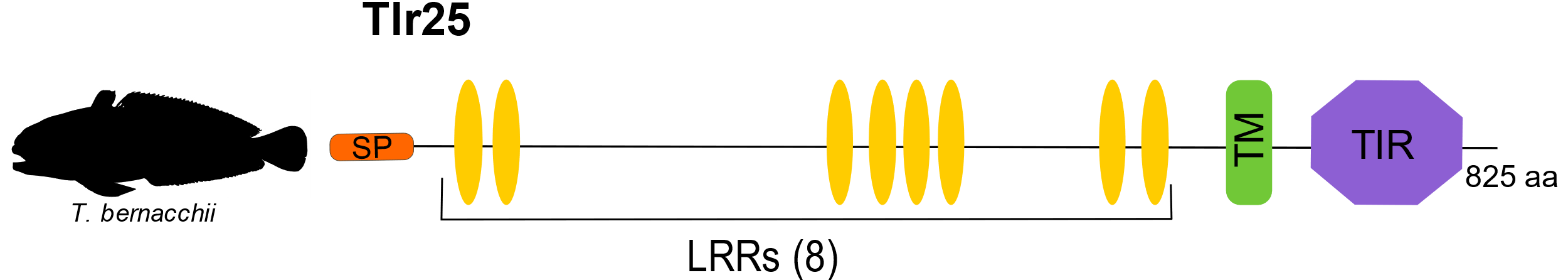


|  |  | **SP** | **LRR** | **TM** | **TIR** | **Size (aa)** |
| --- | --- | --- | --- | --- | --- | --- |
| **Nototheniidae** | *P. georgianus* | P | 7 | 1 | 1 | 778* |
|  | *G. acuticeps* | ni | 7 | 1 | 1 | 825 |
|  | *D. mawsoni* | P | 8 | 1 | 1 | 739* |
|  | *C. gobio* | P | 8 | 1 | 1 | 826 |
|  | *D. labrax* | P | 7 | 1 | 1 | 827 |

**Supplementary Figure 9. The protein domain structure of Tlrs in Nototheniidae.** The deduced structure of the Tlr proteins in the Antarctic teleost *T. bernacchii* are represented as an example of the structure across the Nototheniidae fish. For *tlr23*, which is missing from *T. bernacchii* the structure of the deduced protein in *P. georgianus* was used. The numbers of domains predicted in other Nototheniidae species as well as in the sub-Antarctic fish *C. gobio* and the evolutionary proximate Perciform *G. aculeatus* are indicated in a table (except for *tlr25* where *D. labrax* is represented). Domains represented are: LRR- Leucine rich motifs (yellow), TM- transmembrane domain (green), TIR, Toll/interleukin-1 receptor (purple). The presence of a signal peptide (SP) is also indicated (orange). LRR and TIR were predicted *in silico* using ScanProsite (de Castro et al., 2006) and SMART (Letunic, Khedkar, & Bork, 2021) programmes. The signal peptide (SP) was predicted using the SignalP 4.1 Server (Nielsen, 2017) and TM domains were predicted using the TMHMM Server v. 2.0 (http://www.cbs.dtu.dk/services/TMHMM/). The length (aa) of the predicted proteins is indicated. Sequence alignments mapping all the predicted domains are available in Supplementary Figure 4-8D. Tlr21 (197 aa) from *D. mawsoni* and Tlr25 (369 aa) from *N. coriiceps* are not represented as they are very incomplete. * Partial sequences, P- predicted.

**Supplementary Figure 10. Multiple sequence alignments and sequence annotation of the Nototheniidae TLR1 superfamily members*.* A) Tlr1, B) Tlr2_a, C) Tlr2_b, D) Tlr14/18, E) Tlr25.** Leucine-rich repeat (LRR) are highlighted in yellow and the consensus LRR motifs (LxxLxLxxNxL) is marked in bold; Transmembrane (TM) domain is highlighted in blue and Toll/IL-1 receptor (TIR) is marked in green. Protein domains were predicted using SMART, ScanProsite and TMHMM webservers and by sequence homology. The protein signal-peptide when predicted are highlighted in pink and amino acids coloured in red were found to be under positive selective pressure.

**Supplementary Figure 11. Multiple sequence alignments and sequence annotation of the Nototheniidae TLR3 superfamily members.** Leucine-rich repeat (LRR) are highlighted in yellow and the consensus LRR motifs (LxxLxLxxNxL) is marked in bold; Transmembrane (TM) domain is highlighted in blue and Toll/IL-1 receptor (TIR) is marked in green. Protein domains were predicted using SMART, ScanProsite and TMHMM webservers. The protein signal-peptide when predicted are highlighted in pink. Amino acids coloured in red were found to be under positive selective pressure.

**Supplementary Figure 12. Multiple sequence alignments and sequence annotation of the Nototheniidae TLR5 superfamily members. A) Tlr5 and B) Tlr5S.** Leucine-rich repeat (LRR) are highlighted in yellow and the consensus LRR motifs (LxxLxLxxNxL) is marked in bold; Transmembrane (TM) domain is highlighted in blue and Toll/IL-1 receptor (TIR) is marked in green. Protein domains were predicted using SMART, ScanProsite and TMHMM webservers. The protein signal-peptide when predicted are highlighted in pink. Amino acids coloured in red were found to be under positive selective pressure.

**Supplementary Figure 13. Multiple sequence alignments and sequence annotation of the Nototheniidae TLR7 superfamily members. A) Tlr7, B) Tlr8 and C) Tlr9.** Leucine-rich repeat (LRR) are highlighted in yellow and the consensus LRR motifs (LxxLxLxxNxL) is marked in bold; Transmembrane (TM) domain is highlighted in blue and Toll/IL-1 receptor (TIR) is marked in green. Protein domains were predicted using SMART, ScanProsite and TMHMM webservers. The protein signal-peptide when predicted are highlighted in pink. Amino acids coloured in red were found to be under positive selective pressure.

**Supplementary Figure 14. Multiple sequence alignments and sequence annotation of the Nototheniidae TLR11 superfamily members. A) Tlr13, B) Tlr21, C) Tlr22 and D) Tlr23.** Leucine-rich repeat (LRR) are highlighted in yellow and the consensus LRR motifs (LxxLxLxxNxL) is marked in bold; Transmembrane (TM) domain is highlighted in blue and Toll/IL-1 receptor (TIR) is marked in green. Protein domains were predicted using SMART, ScanProsite and TMHMM webservers. The protein signal-peptide when predicted are highlighted in pink. Amino acids coloured in red were found to be under positive selective pressure.


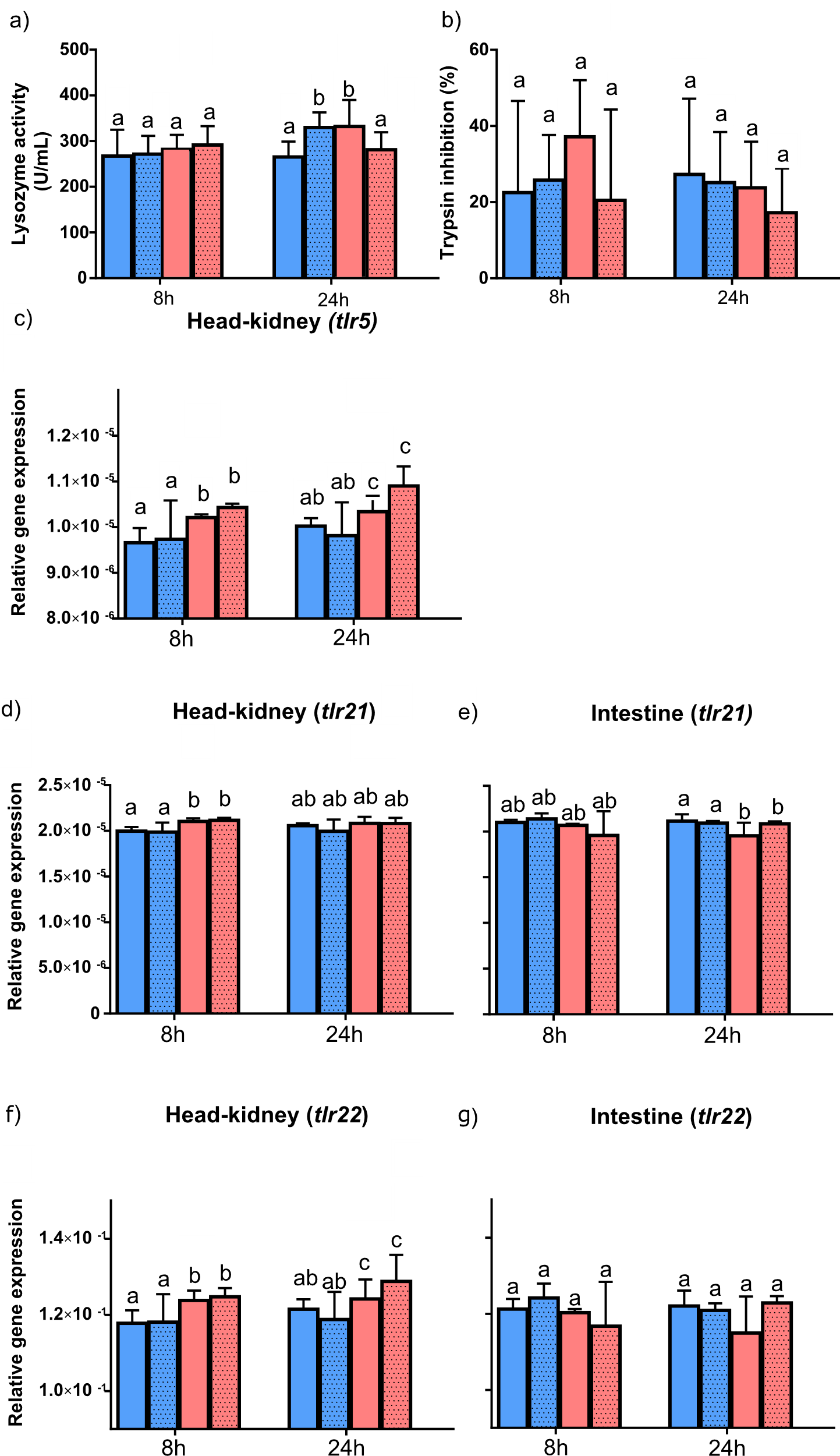


**Supplementary Figure 15.** **Quantitative expression** **(ng/µl) of *tlr5, tlr21* and *tlr22* (A) and plasma immune-related enzyme activity (B) in LPS and temperature challenged *N. rossii*. A)** Gene transcript abundance was analysed in the head-kidney and intestine of control (clear bars) and the LPS (dotted bars) experimental groups at 2ºC (blue bars) and 6ºC (red bars) and by time post-injection (8h and 24h). Expression of *tlr5* in the intestine is of very low abundance and could not be quantified. Data corresponds to the mean ± SEM of six different samples per group and gene expression levels were normalized using the geometric mean of two reference genes (*18s* and *β-actin*). SigmaPlot software v12.5 was used to identify significant differences between the experimental groups using three - way analysis of variance (ANOVA) and a post hoc Tukey´s test (Shapiro-Wilk normality test). Significant differences are indicated with an asterisk (p < 0.05) or a double asterisks (p < 0.001). B) Lysozyme (U/ml) and total protease inhibition (%) were measured in plasma from control (clear bars) and the LPS (dotted bars) experimental groups at 2ºC (blue bars) and 6ºC (red bars) and at 8h and 24h post-injection. Data corresponds to the mean ± SEM of six different samples per group. The concentration of total plasma proteins was similar in all experimental groups (data not shown). SigmaPlot software v12.5 was used to identify significant differences between the experimental groups using three - way analysis of variance (ANOVA) and post hoc Tukey´s test (Shapiro-Wilk normality test). Significant differences are indicated with asterisks and letters (p < 0.05).

**Supplementary Table 1.** List of species name, abbreviations, accession numbers and database source. The sequences that were not used for the phylogenetic tree are indicated. This file is available in excel file.

**Supplementary Table 2. qPCR primer pairs, product size, annealing temperatures and efficiency.**

|  | Sequences (5`🡪3`) | bp | T (ºC) | Efficiency (%) |
| --- | --- | --- | --- | --- |
| *tlr5* Fw | *TTTCGTCCAGAGGAGGGAGT* | 168 | 55 | 90.4 |
| *tlr5* Rv | *TTGGTCCGATGTTCTCCAGC* |  |  |  |
| *tlr21* Fw | *TGGTGGAAATGTGAAGTATGATCC* | 87 | 55 | 90.5 |
| *tlr21* Rv | *AGCACATCAATTAAATGACACCTCT* |  |  |  |
| *tlr22* Fw | *AGTTTCACCTGTGACTGCGA* | 87 | 55 | 90.4 |
| *tlr22* Rv | *AAAGTTGGAGGCGTCAACCA* |  |  |  |
| *18S* Fw | *TGACGGAAGGGCACCACCAG* | 158 | 58 | 93.6 |
| *18S* Rv | *AATCGCTCCACCAACTAAGAACGG* |  |  |  |
| *β-actin* Fw | *AACCCAAACGACTGGCTCTG* | 174 | 58 | 90 |
| *β-actin* Rv | *TTCCACACATTCACACCGCA* |  |  |  |

**Supplementary Table 3. TLRs identified in fish and other vertebrates.** The table summarizes the outcome of the BI phylogenetic tree. The Nototheniidae and other fish Tlrs were classified according to sequence clustering. Lamprey sequences were not included.


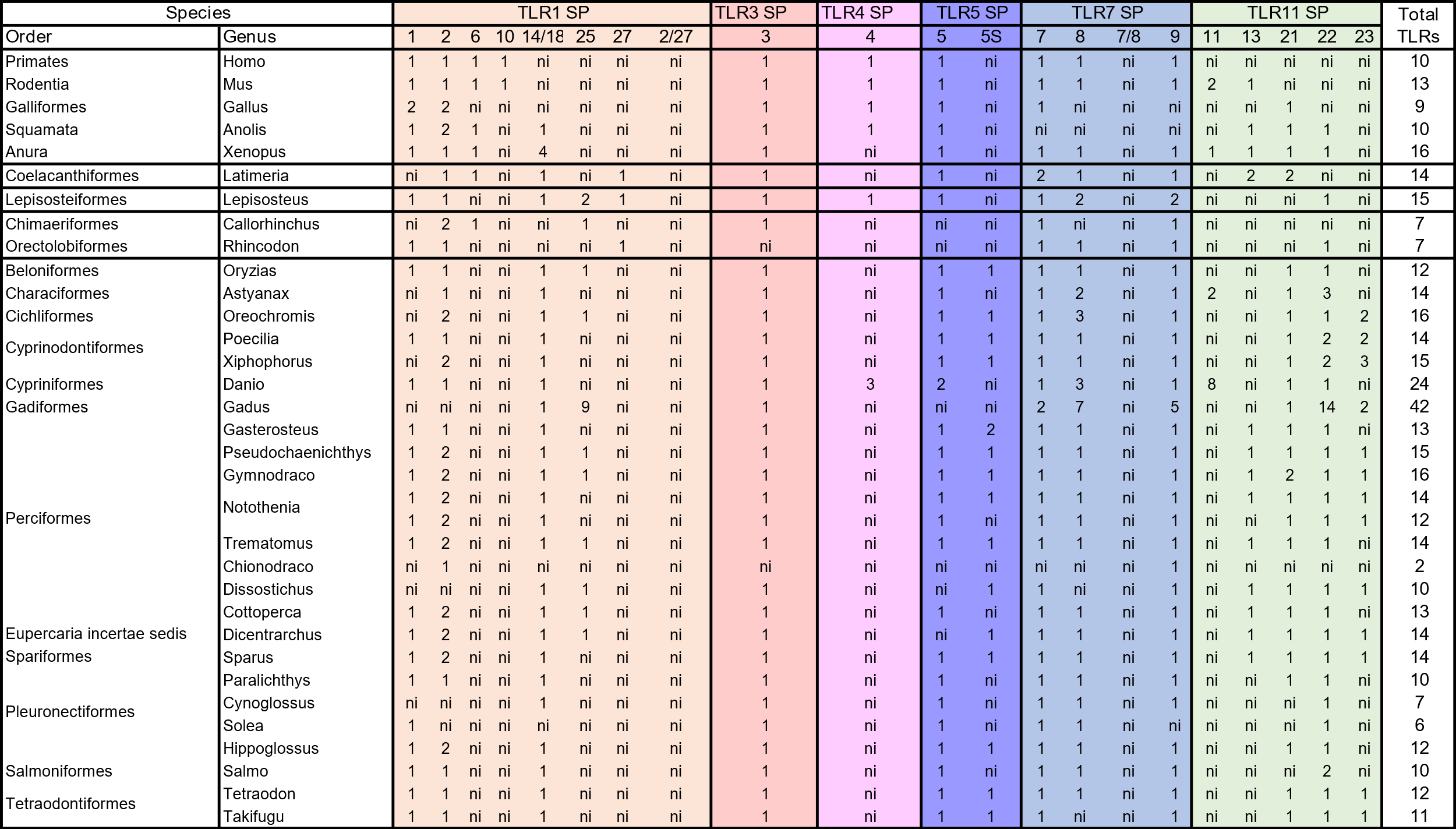


**Supplementary Table 4.** Percent (%) of amino acid sequence identity between the Nototheniidae A) *tlr1*, B) *tlr2*, C) *tlr3*, D) *tlr5*, E) *tlr5S*, F) *tlr7*, G) *tlr8*, H) *tlr9*, I) *tlr13*, J) *tlr14/18*, K) *tlr21*, L) *tlr22*, M) *tlr23* and N) *tlr25* subfamilies and with other Perciformes fish.

a)


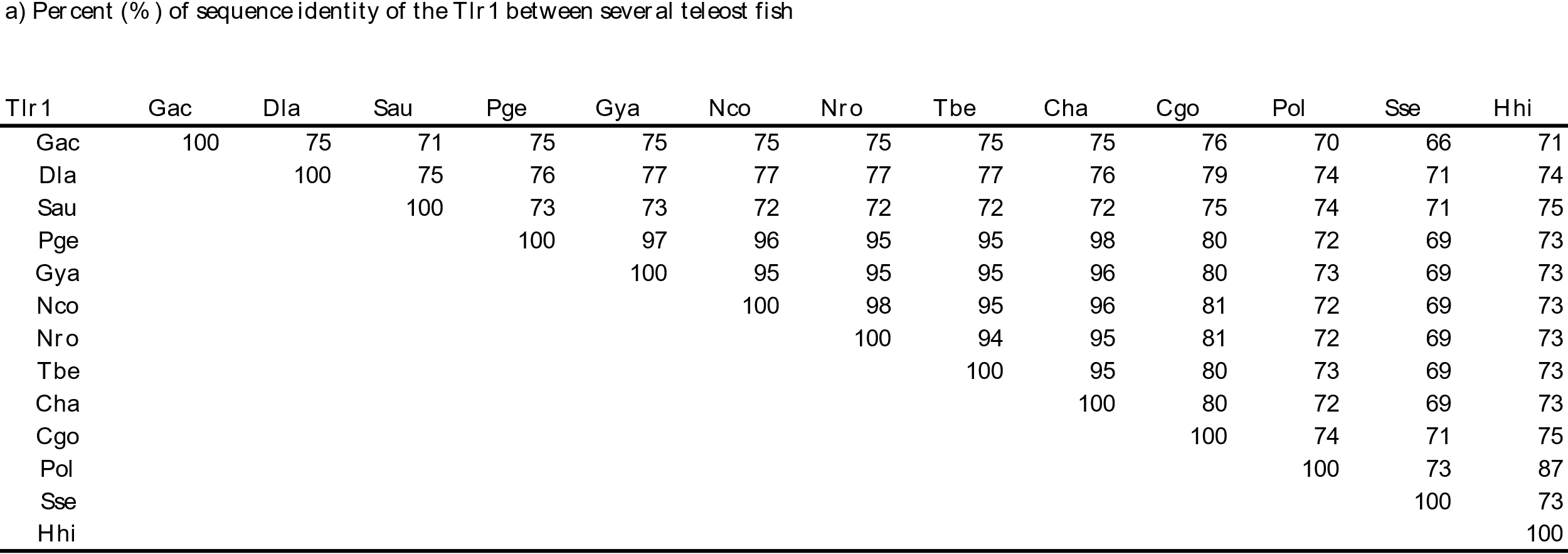


b)


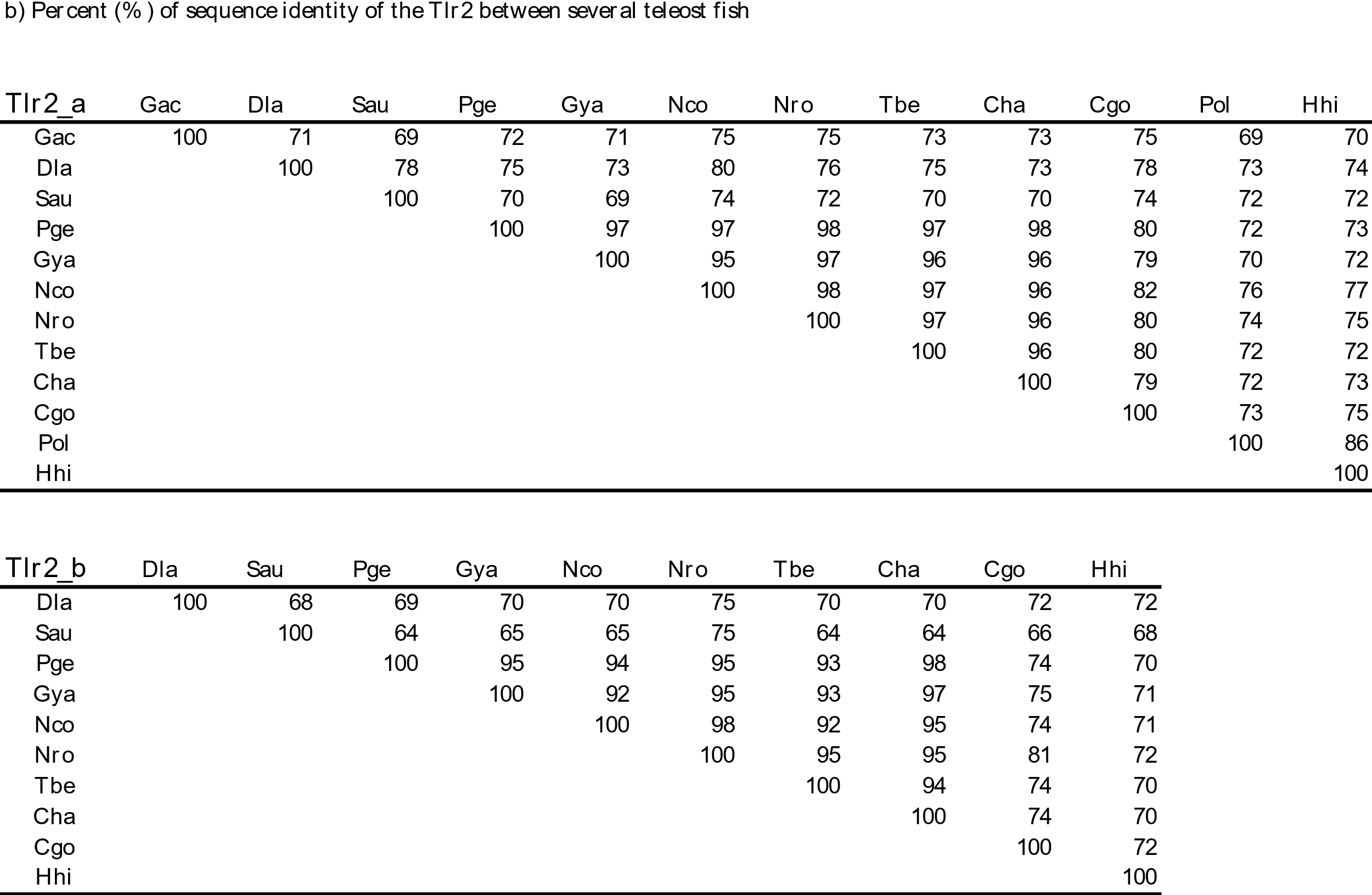


c)


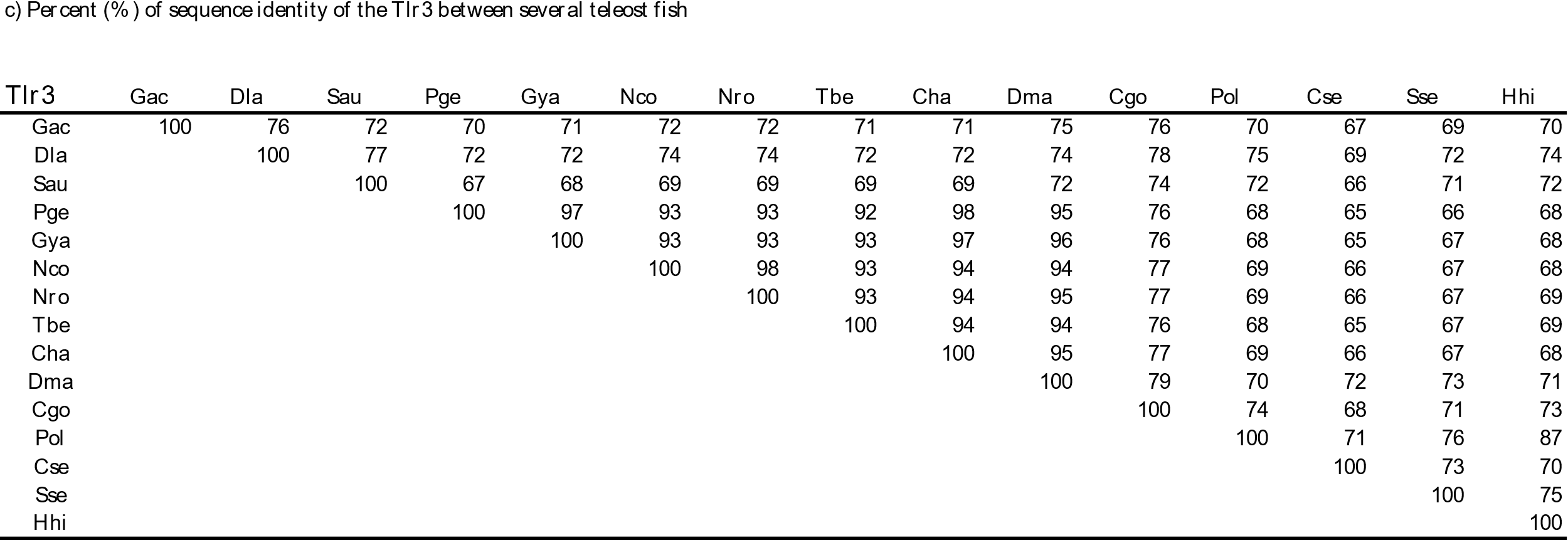


d)


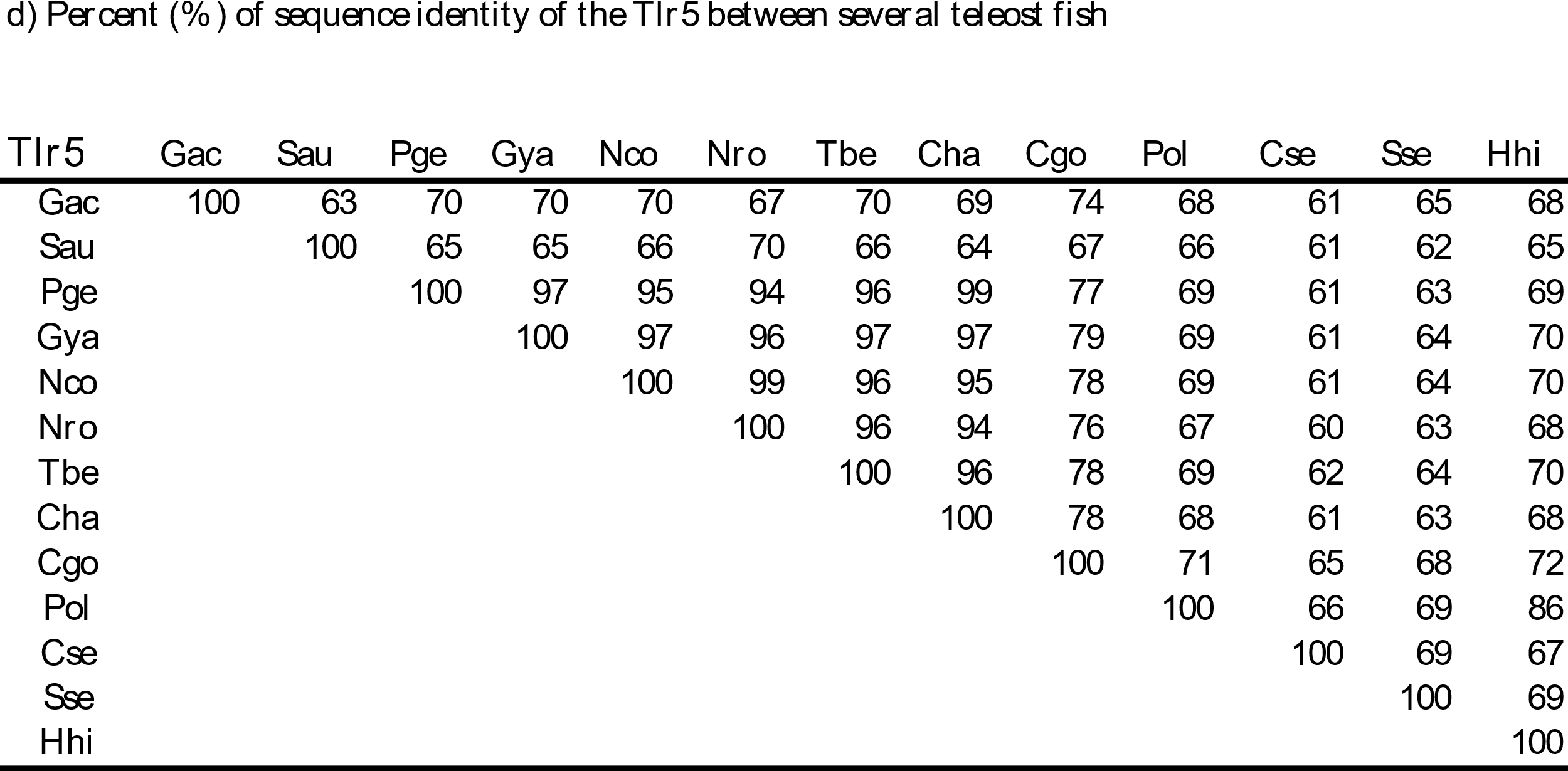


e)


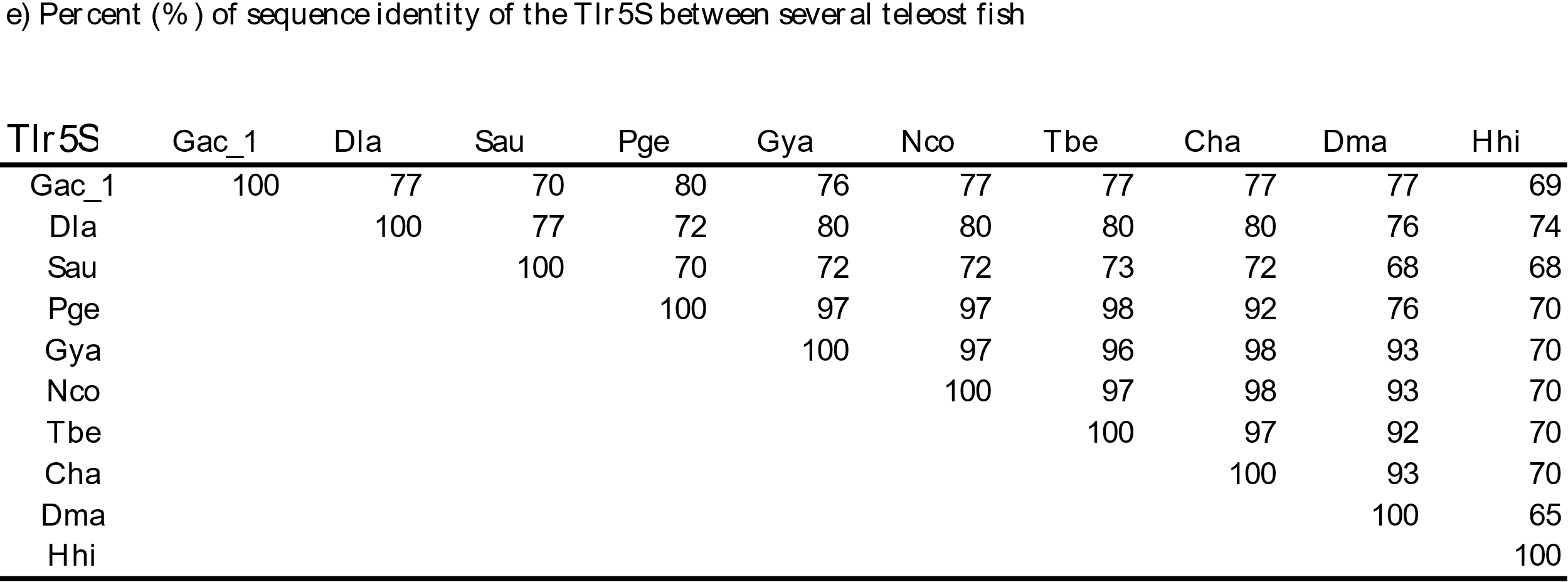


f)


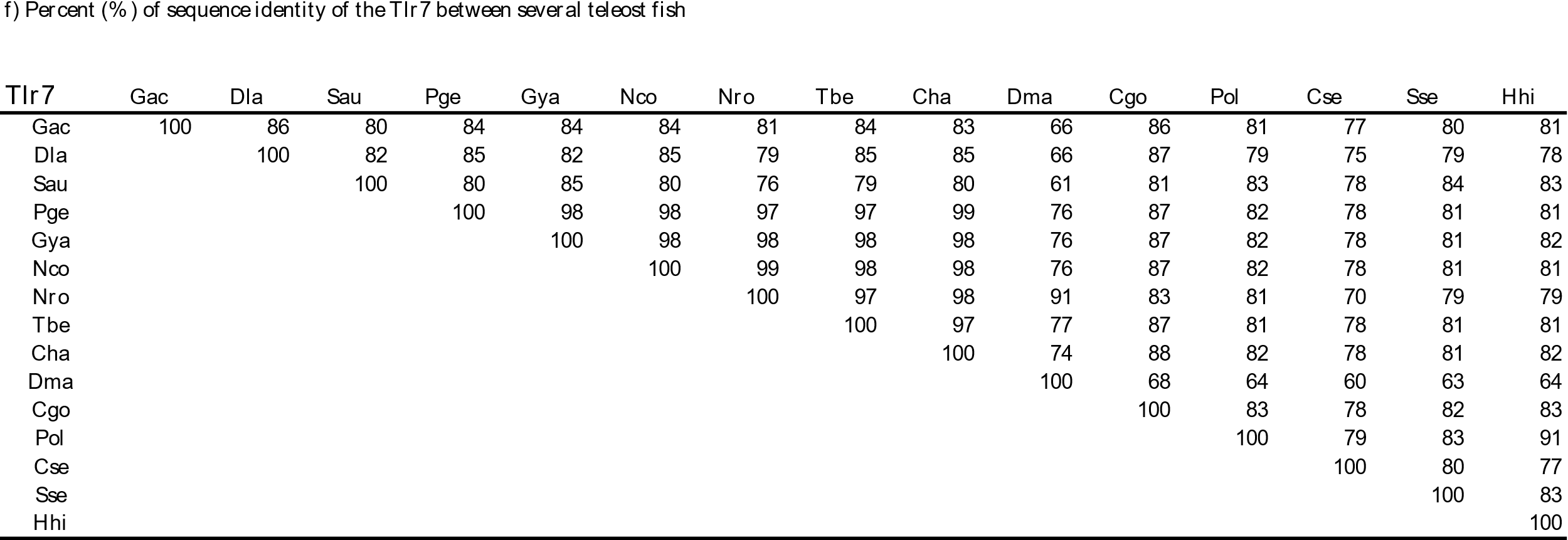


g)


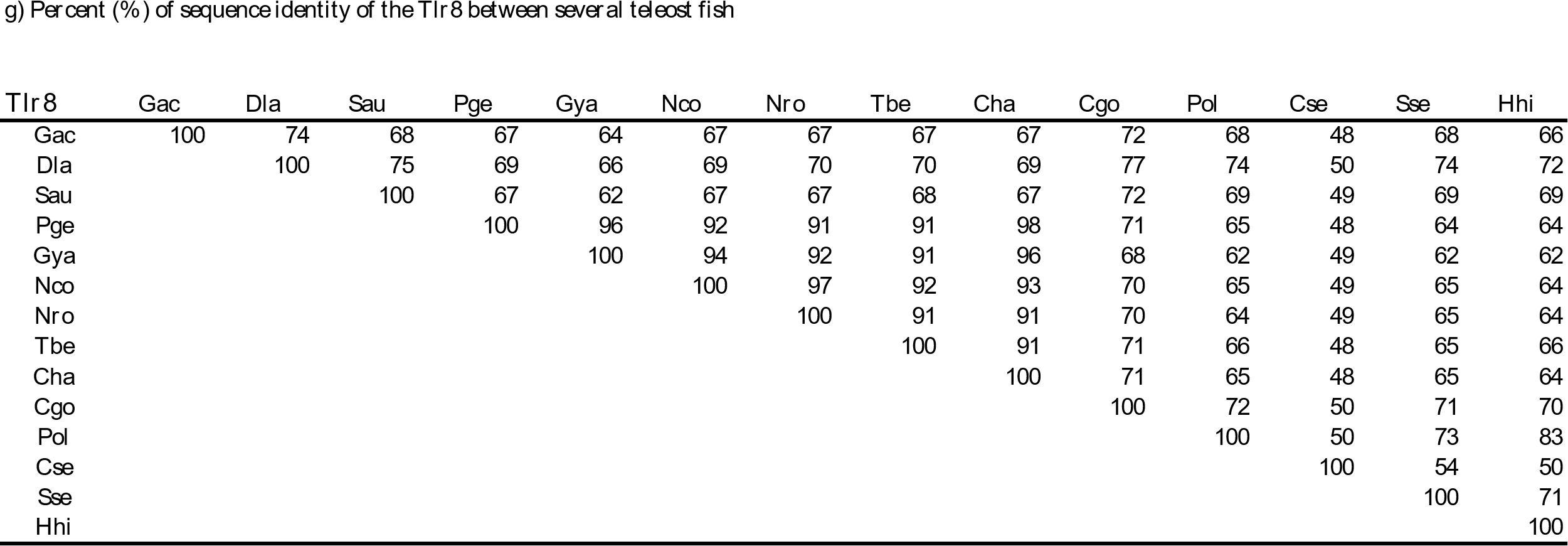


h)


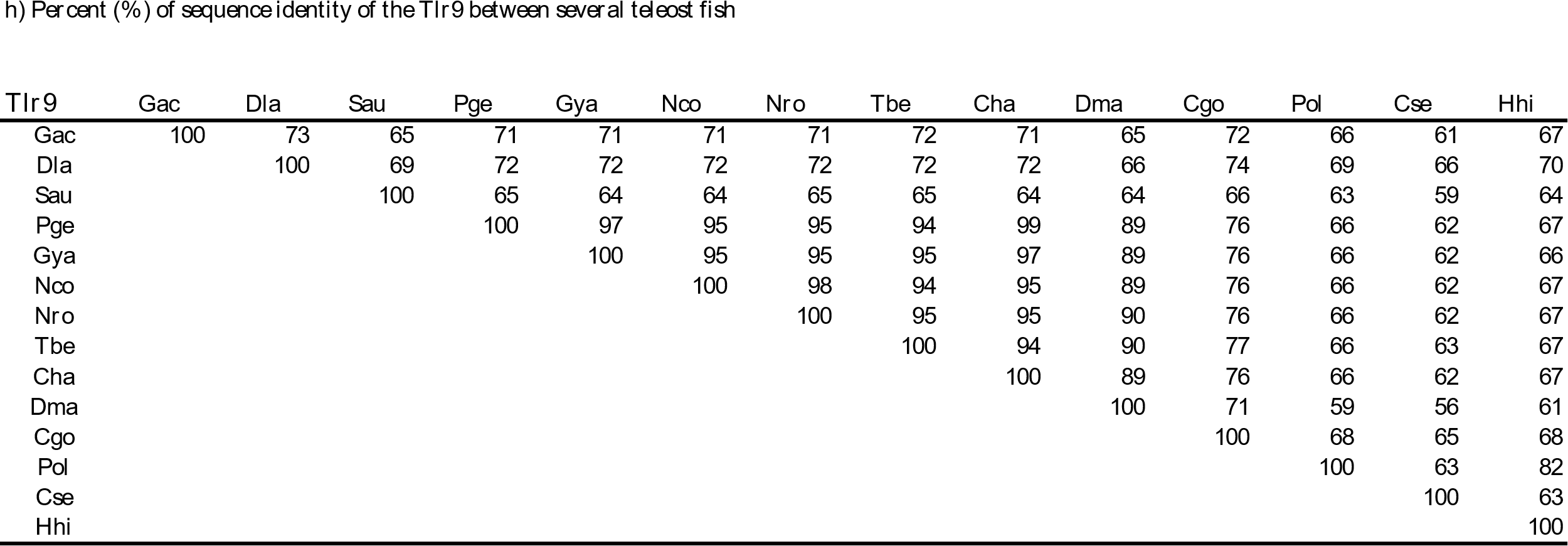


i)


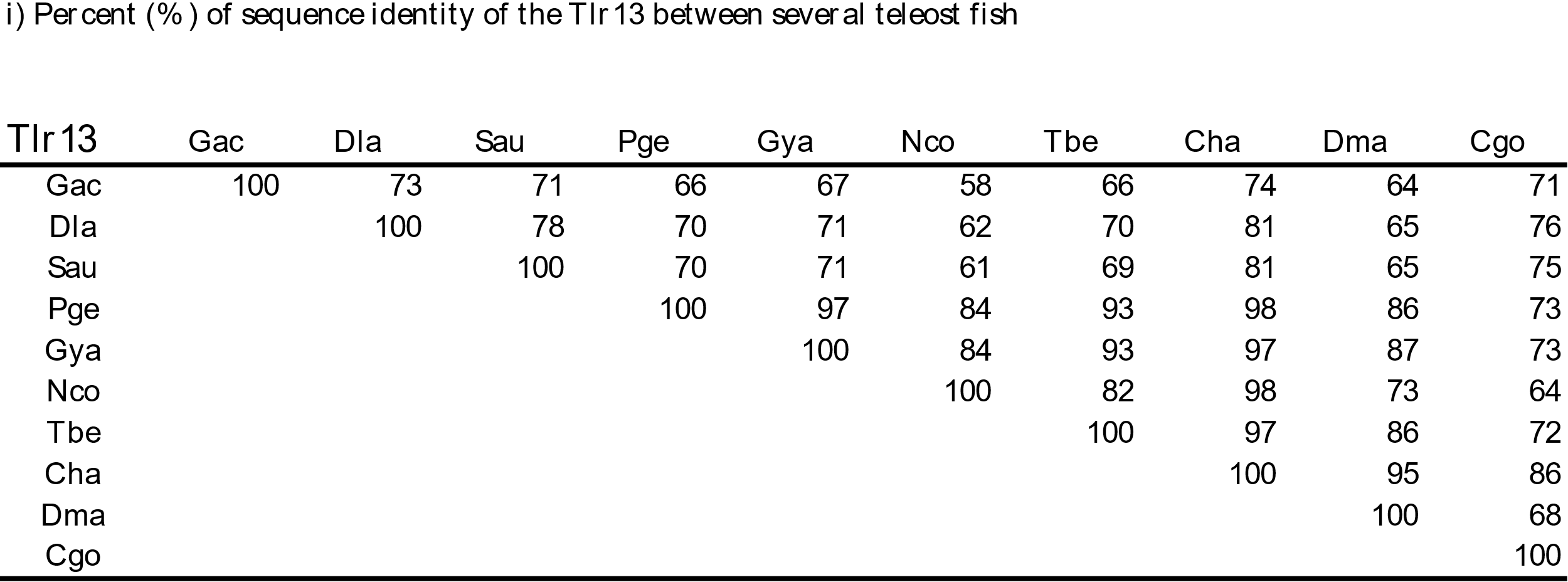


j)


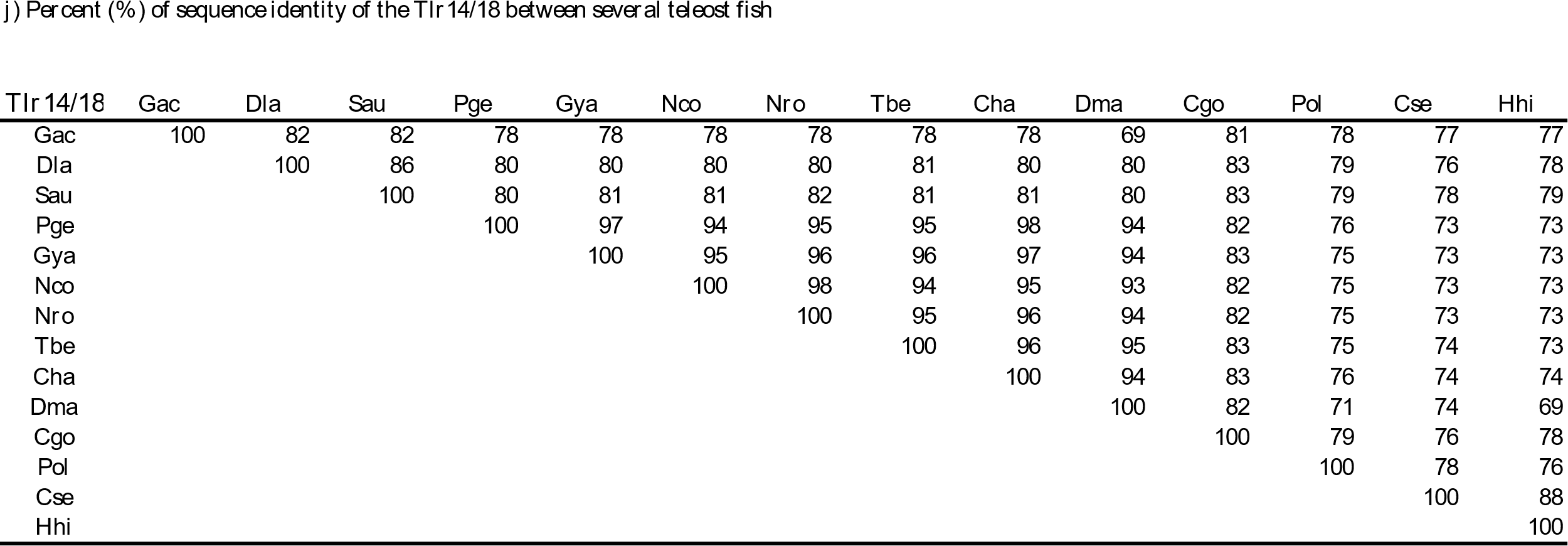


k)


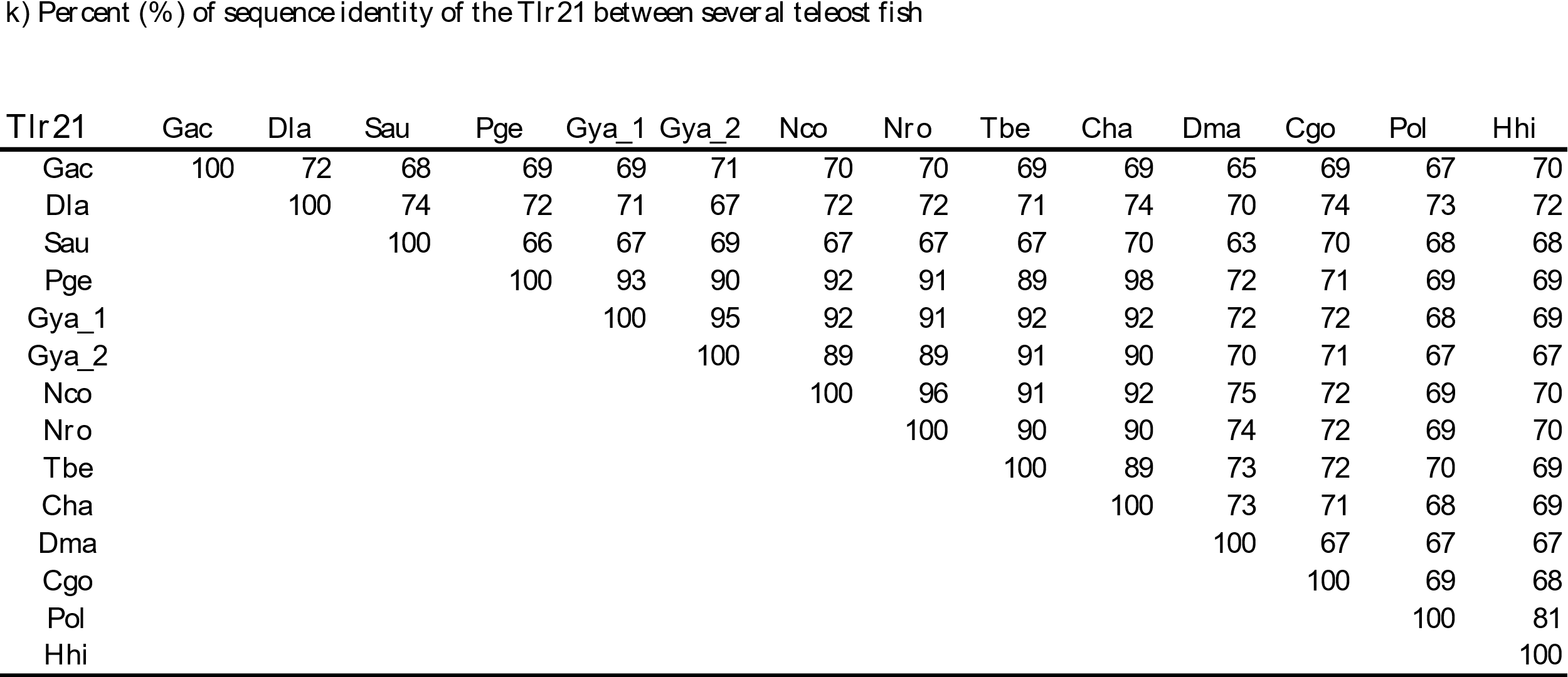


l)


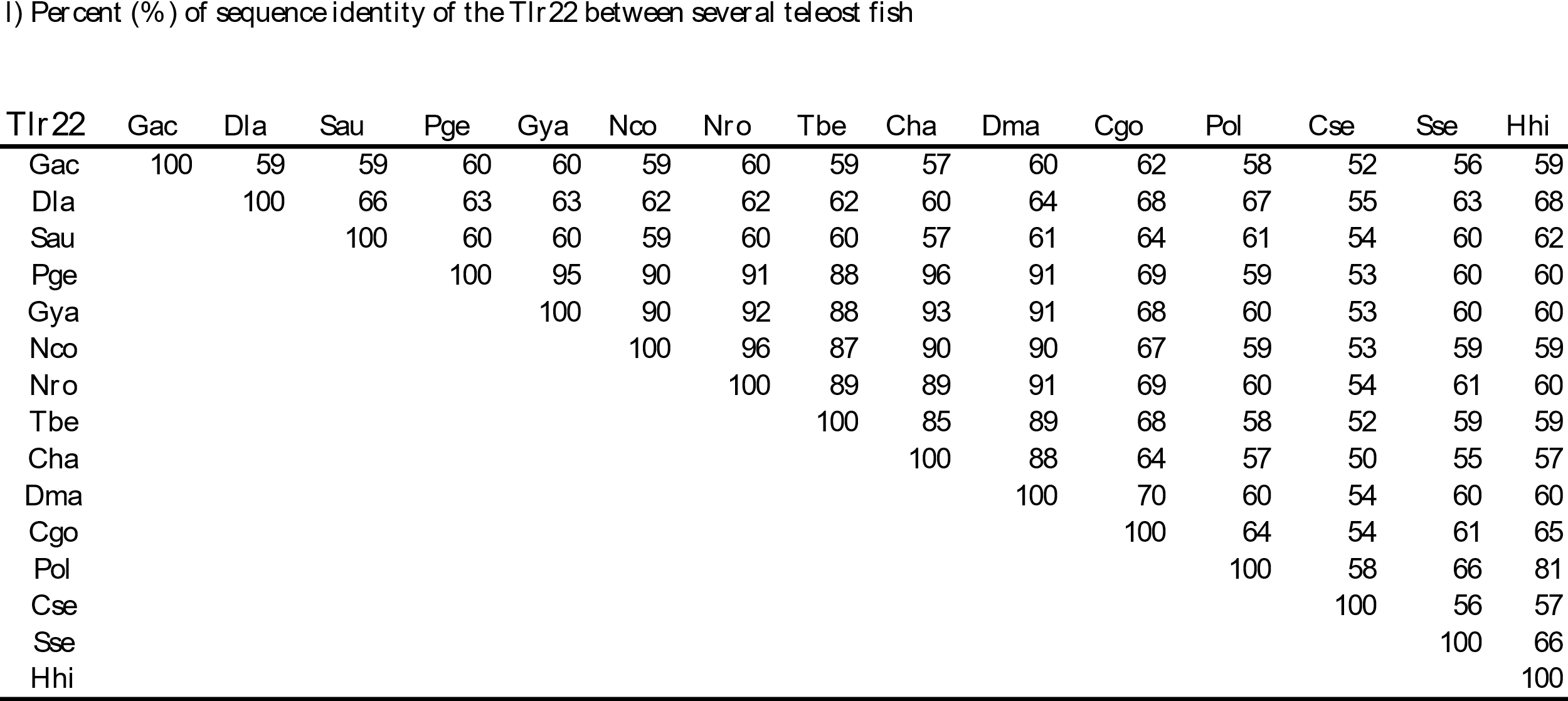


m)


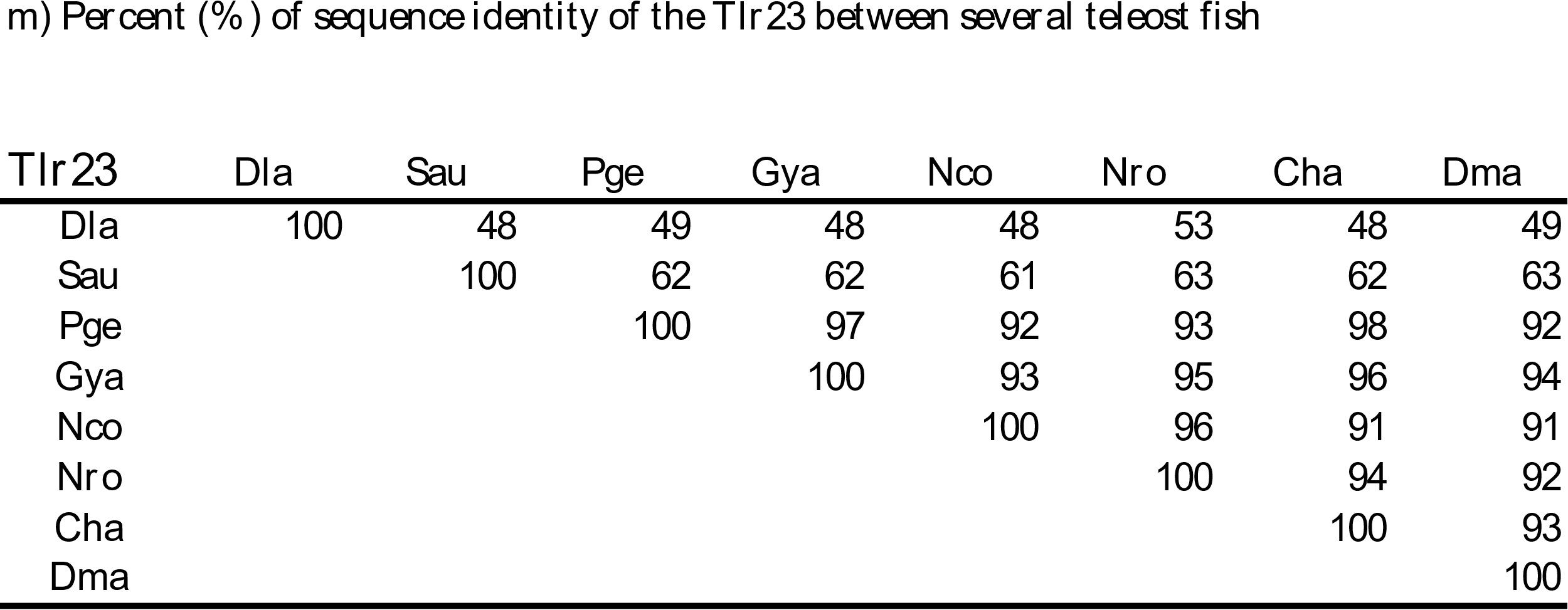


n)


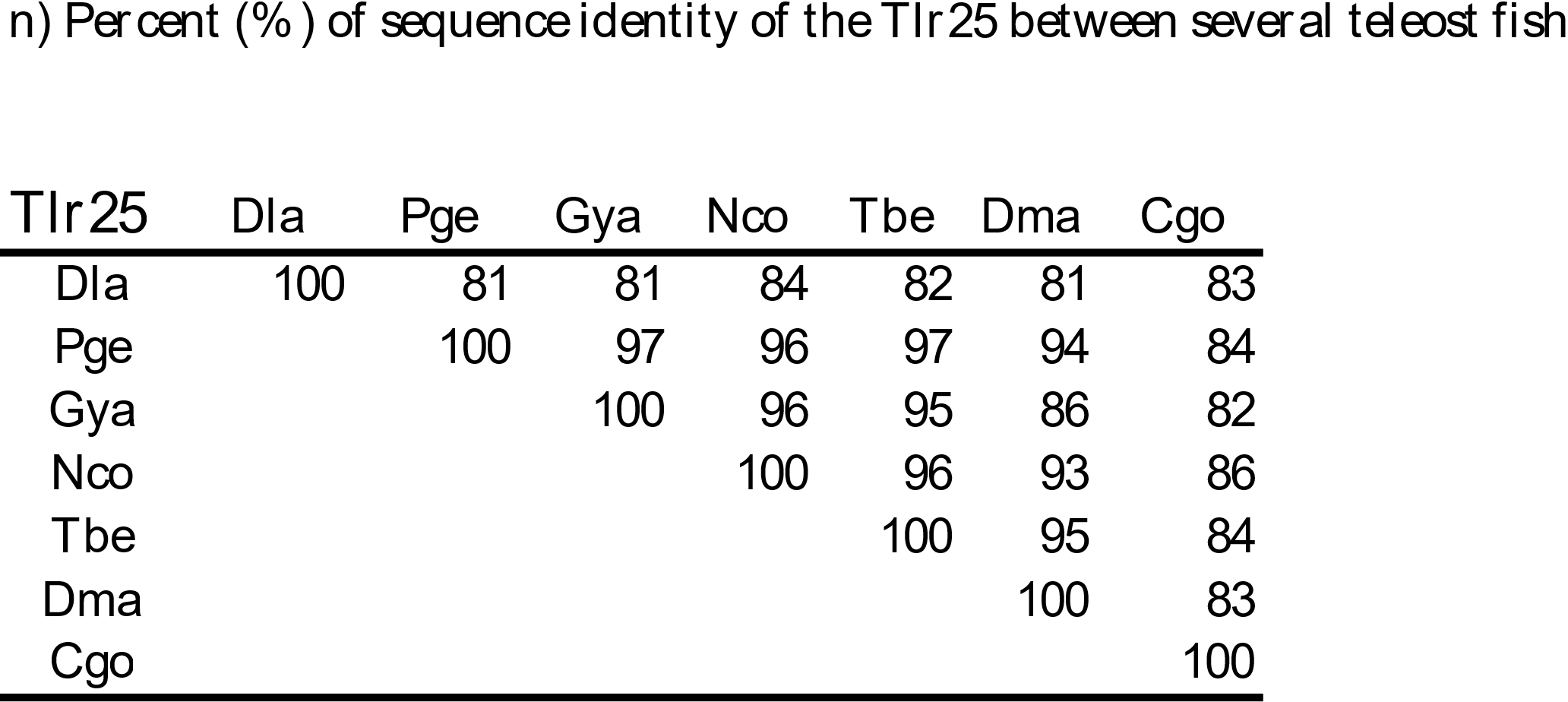

Supplement: Supplementary file 1 [file DataSheet_1.docx]
